# All‐in‐One Structured Lithium‐Metal Battery

**DOI:** 10.1002/advs.202200547

**Published:** 2022-04-13

**Authors:** Lei Dong, Chang Zhang, Wei Liu

**Affiliations:** ^1^ School of Physical Science and Technology ShanghaiTech University Shanghai 201210 China

**Keywords:** all‐in‐one structure, fast ion transport kinetics, high‐performance, lithium‐metal batteries, microcell arrays

## Abstract

3D batteries possess apparent advantage in electrochemical ion‐transport kinetics than the conventional‐structured batteries. However, due to the special electrode configuration and fabrication complexity, 3D battery design has inherent issue of mechanical stability and only succeeds in microsystems, far from ideal. Herein, a high‐stable, all‐in‐one structured 3D lithium‐metal battery is designed which consists of paralleled microcell arrays. Fast ion‐transport kinetics in full cell level can not only address the key issue of lithium dendrites in anode but also improve electrochemical performance of cathode. As a result, the resultant lithium metal anode acquires long‐term stability of 1000‐cycle life at a high current density of 10 mA cm^−2^. Also the all‐in‐one structured lithium metal battery has general applicability for various cathodic materials and delivers significantly improved rate and cycling performance, as well as high areal capacity up to 10.4 mAh cm^−2^.

## Graphical abstract

All‐in‐one structure is designed for stable, high‐performance lithium‐metal battery. The formed paralleled microcell array performs high mechanical stability and facilitates fast ion‐transport kinetics in full cell level, which not only addresses the dendrites issue of lithium metal anode but also improves electrochemical performance of cathode.

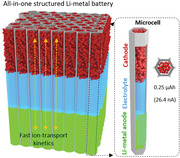

## 1 Introduction

Structural engineering has proved its indispensability in constructing high‐performance electrochemical electrodes for batteries,^[^ ^1^, ^2^ ^]^ supercapacitors,^[^ ^3^, ^4^ ^]^ catalysts,^[^ ^5^, ^6^ ^]^ fuel cells,^[^ ^7^, ^8^ ^]^ etc. With control of pores, surface chemistry, crystal structure, stacking manner and morphology of active particles, the structure of electrode can be well designed on micro‐ or nanoscale, which facilitate reaction kinetics and therefore boost electrochemical performance of active materials. For Li‐ion batteries (LIBs), both anode and cathode often perform worse in capacity, rate and cycling stability than their theoretical limits,^[^ ^9^, ^10^, ^11^, ^12^, ^13^ ^]^ especially for high loading mass or in high rate.^[^ ^14^, ^15^ ^]^ This is because the irregularly shaped active particles tend to stack in disordered manner, giving obstructed or tortuous interparticle channels and thereby leading sluggish charge transport kinetics. Tremendous effort has been devoted in order to create aligned channels with low tortuosity in electrodes.^[^ ^15^, ^16^, ^17^, ^18^, ^19^ ^]^ For example, strategies inducing vertical alignment of active materials can improve the rate performance and also increase the areal loading mass of active materials.^[^ ^1^, ^16^, ^20^, ^21^ ^]^ However, the major difficulty lies in creating well‐defined, continuous ion‐transport channels while maintaining high structural stability from volume fluctuations of the active particles.

Structural‐integrated three‐dimenstional (3D) battery design provides an alternative solution to enhance ion‐transport kinetics by integrated spatial electrode architecture.^[^ ^22^, ^23^, ^24^, ^25^, ^26^, ^27^ ^]^ For example, 3D interdigitated batteries bringing anode/cathode into close proximity not only shorten ion‐transport pathway but also increase electrolyte–electrode interface, therefore could maximize areal energy and power densities at full‐cell level.^[^ ^23^ ^]^ Sun et al. demonstrated that 3D‐printed Li_4_Ti_5_O_12_‐LiFePO_4_ microbattery with interdigitated architecture enabled electrode height over 200 µm and as expected, delivered higher areal energy and power densities than the traditional 2D batteries prepared by slurry coating.^[^ ^28^ ^]^ However, such 3D electrode configuration relies on complex procedures and exotic fabrication techniques (e.g., 3D‐printing, photoetching, electroplating, et al.).^[^ ^29^, ^30^ ^]^ Also the 3D morphology and interdigitated architecture arouses a big concern of mechanical instability. A template‐based strategy may improve the structural stability by integrating anode and cathode in channels of anodic aluminum oxide membrane^[^ ^31^ ^]^; however, this is at the cost of low active/inactive component ratio and reduced areal capacity, as well as complex technique for coating electrodes.

Li metal batteries (LMBs) have been placed great expectation toward next‐generation, high density energy storage for electric vehicles (EVs) and smart grid due to the ultrahigh theoretical specific capacity and the lowest electrochemical potential of Li metal anode.^[^ ^32^, ^33^, ^34^, ^35^, ^36^, ^37^ ^]^ However, unlike the traditional graphite anode, highly active Li metal is linked with serious safety issue that Li dendrites form and keep growing during electrochemical cycles, which results in unstable solid electrolyte interphase (SEI) layer degrading battery performance and even impales separator, triggering safety hazard.^[^ ^38^, ^39^, ^40^ ^]^ Likewise, structural engineering has demonstrated some success in achieving “dendrite‐free” Li anode by various techniques including 3D frameworks, artificial SEI layer, protective film, etc.^[^ ^20^, ^41^, ^42^, ^43^, ^44^ ^]^ Among them, 3D frameworks can not only homogenize Li‐ion deposition by the inner micro‐/nano‐scale pores, but also physically confine the infinite volume fluctuation of Li metal during Li‐stripping/platting cycles. However, the current achievements are still far from ideal. Electrochemically stable Li metal anode and high‐performance cathode are high desirable, as well as a mechanically stable LMB configuration.

**Figure 1:**
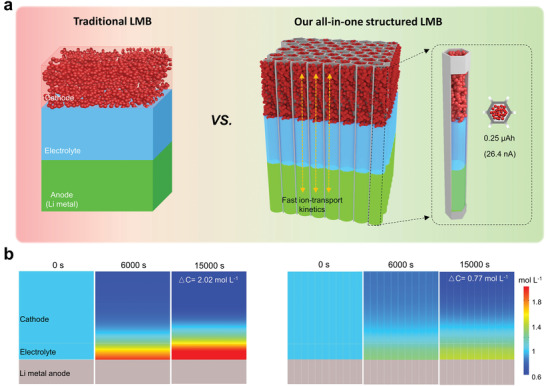
a) Schematic illustration showing the comparison between our all‐in‐one structured LMB and the traditional LMB. The left shows the current value (26.4 nA) and specific capacity (0.25 µAh) per microcell for a LMB with LFP loading mass of 76 mg cm^−2^. b) Simulation of ion‐transport kinetics in the traditional three‐layered LMB with separator membrane (left) and our structurally integrated LMB (right). Local Li‐ion concentration evolves along with electrochemical reaction time (1 mA cm^−2^ of current density).

**Figure 2:**
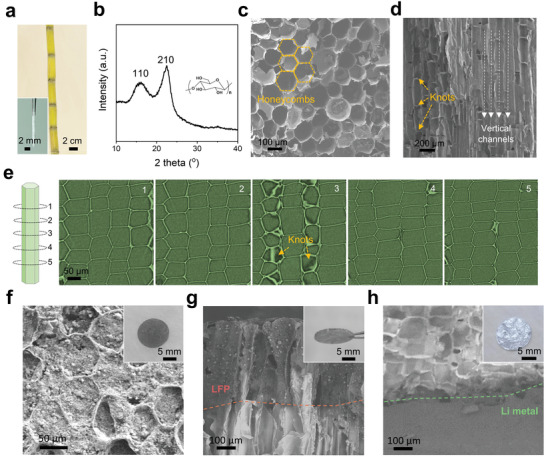
a) Photos of sugarcanes and one sugarcane chip (inset). b) XRD spectrum of sugarcane after washed by water and ethanol. c) From top‐view SEM image, sugarcane shows honeycomb structure with unit size ranging from 40 to 110 µm. d) Sectional‐view SEM image of sugarcane show ordered, vertically aligned microchannels sectioned by the knots. e) 2D XTM virtual slices of sugarcane display morphological evolution along the channel direction, demonstrating its structural model of paralleled microtube arrays. The left shows the corresponding structural model. f–h) SEM images show the embedded commercial LFP cathode f,g) and Li metal anode h). Insets are the corresponding optical photos.

Herein, we designed an all‐in‐one structured LMB in order to improve the performance of cathode and Li metal anode. All the components including Li‐metal anode, liquid electrolyte and cathodic materials are embedded into the microtubes of one sugarcane‐derived framework (**Figure** **1** and Figure S1, Supporting Information), forming paralleled, microcell array, which differs from the traditional three‐layered LMB. The continuous, vertically aligned microchannels in natural sugarcane offer direct, non‐tortuous ion‐transport pathways across the whole electrochemical system (**Figure** **2**a–d), which not only homogenize Li‐ion flux and confine the volume fluctuation of Li metal but also facilitate ion‐transport kinetics in cathode. Via a simple, self‐absorption process, various cathode materials including LiCoO_2_ (LCO), LiFePO_4_ (LFP) and LiNi_0.8_Co_0.1_Mn_0.1_O_2_ (NCM811) can be coated directly in high areal mass loading. Note that due to the ultrahigh porosity (92.5–96.8%) but strong mechanical stability of sugarcane framework, inactive component in device maintains in low mass fraction (<20 wt%). As expect, the resultant LiFePO_4_‐based LMB showed high areal energy density of 10.4 mAh cm^−2^ at current density of 1.2 mA cm^−2^, as well as improved rate performance and cycling stability in comparison with the traditional battery counterparts.

## 2 Results and Discussion

The goal of this work is to create a simple, reliable structure for high‐performance, stable LMB where both cathode and Li metal anode are well built. As mentioned above, vertically aligned structure has long been seen as an ideal model which can minimize geometric tortuosity (*τ*) and thus improves the effective Li‐ion diffusion coefficient (*D*_Li+_), according to the equation $\tau \;=\;\varepsilon \frac{D_{0}}{D_{\mathrm{Li}+}}$, where *D*_0_ and *ε* is intrinsic Li‐ion diffusion coefficient and volume fraction of conductive phase, respectively.^[^ ^14^, ^15^, ^16^ ^]^ The resultant fast Li‐ion transfer capability behaves in high ion conductivity (*σ*) in cell, as illustrated in Figure S2, Supporting Information.^[^ ^20^, ^21^ ^]^ Here, electrochemical kinetics of LMBs was simulated to prove the architectural advantage of our all‐in‐one structure in comparison with the traditional three‐layered structure (Figure 1b,c and Figure S3, Supporting Information). Evolution of Li‐ion concentration was calculated in electrolyte layer and the internal electrode during electrochemical reaction with current density of 1 mA cm^−2^ and an initial Li‐ion concentration of 1 m. In Figure 1b,c, the color mappings represent local Li‐ion concentration and the differential concentration between the top of electrolyte layer and bottom of cathode materials reflects the corresponding Li‐ion transport kinetics, noted as △C. Due to the vertical honeycomb channels, our all‐integrated model exhibits smaller △C than the common LMB during the all electrochemical process. Such low concentration polarization facilitates fast electrochemical reaction kinetics, especially for thick cathodes. As expect, the simulated voltage evolution shows higher discharging plateau for the structural‐integrated architecture (Figure S3, Supporting Information), which is advantageous to the specific capacity.

The structure and morphology of sugarcane were first characterized by optical photo (Figure S4, Supporting Information), X‐ray diffraction (XRD, Figure 2b), scanning electron microscopy (SEM, Figure 2a–d and Figure S5, Supporting Information) and X‐ray photoelectron spectroscopy (XPS, Figure S6, Supporting Information). In Figure 2b, sugarcane displays typical cellulose peaks around 15.8^o^ and 22.6^o^, corresponding to (110) and (200) plane respectively.^[^ ^45^ ^]^ This accords with the previous report that about 65–85% of sugarcane bagasse consist of nanocellulose.^[^ ^46^ ^]^ Also XPS spectrum demonstrated its functional groups of C‐C and C‐O bonds (Figure S6, Supporting Information). The honeycomb structure and vertically aligned microchannels can be well observed in Figure 2c,d and Figure S4, Supporting Information. The unit size of honeycomb ranges from 40 to 110 µm and the microchannels show long‐range continuity along the vertical direction. Moreover, the microchannel was sectioned by the several knot layers (≈350 nm in thickness), showing bamboo‐like morphology (Figure 2c and Figure S5, Supporting Information). Such sectioned microstructure offers mechanical stability for sugarcane framework and avoid excessive invasion of cathode or anode materials. Also, X‐ray tomographic microscopy (XTM) was used to show the internal 3D morphology of sugarcane in nondestructive way. Structural evolution was characterized by a series of two‐dimensional (2D) tomographic “slices” along the channel direction (Figure 2e). Honeycombs with thin walls were clearly observed and knots can be identified from the brightness region (3^th^ in Figure 2e), demonstrating the structure of paralleled, hollow microtubes.

The fabrication process of the all‐in‐one structured LMB was illustrated in Figure S1, Supporting Information. Sugarcane was first washed repeatedly and cut into chip as framework. The chip thickness was controlled to be around 1.2 mm in order to load enough active materials and also avoid battery short circuit. Then both the two surfaces were functionalized by large‐sized graphene sheets in order to improve the conductivity of sugarcane framework (LG, ≈62 µm in mean size, based on the method we reported previously^[^ ^47^, ^48^ ^]^) in order to improve electrode conductivity and avoiding internal penetration (Figure S7, Supporting Information). LG dispersion (1.2 mg mL^−1^) was added drop‐wise on surface of dry sugarcane chip. As solvent absorption, graphene sheets were filtered and blocked by the knots in the microchannel (Figure S8, Supporting Information), giving graphene‐functional segment with thickness around 300 µm. Note that the LG loading mass was controlled to be less than 0.09 mg cm^−2^ in order to avoid blocking ion transport. This was proved by the ion conductivity test of sugarcane chip by electrochemical impedance spectroscopy (EIS) method in Figure S9, Supporting Information.^[^ ^16^ ^]^ Then, one surface was further functionalized via filtration of MnO_2_ nanoparticles since the recent works have demonstrated that coated metal oxides can improve the wettability of lithiophobic skeleton.^[^ ^49^, ^50^ ^]^ Similarly, the loading depth of MnO_2_ particles was limited by the knots in microchannels. Then cathode was loaded onto the graphene‐functionalized surface by slurry coating or a self‐absorption process (Figure S10, Supporting Information, see below for detailed discussion). As shown in Figure 2f,g, LFP micron particles combining with binder and conductive black can be filled into the honeycomb channels, as well as other cathodic materials such as LCO and NCM811 (Figure S11, Supporting Information). The amount of slurry determinates areal loading mass of cathodic materials. Then after drying, lithium metal was embedded from the MnO_2_ functionalized surface from the molten state at 250 °C. As expected, MnO_2_ functionalized surface exhibited good wettability of molten lithium, albeit the lithiophobic nature for raw sugarcane (Figure S12, Supporting Information). Li metal can be distinguished from cross‐sectional SEM image and metallic luster was observed in outer surface (Figure 2h). Also the areal loading mass of lithium metal depends on the usage amount of raw Li foil and is restricted in MnO_2_ functionalized region due to the lithiophobic nature of sugarcane (Figure S13, Supporting Information). Here, in order to avoid the possible increase of electrical conductivity by carbonization, conductivity test of sugarcane chip was tested after heat treatment. As shown in Figures S14 and S15, Supporting Information, despite with heat treatment at 400 °C for 10 min, the electrical conductivity of sugarcane chip is only 1.34 × 10^−11^ S cm^−1^, close to an insulator. Finally, liquid electrolyte was injected and wet the whole cell rapidly, as proved in Figure S16, Supporting Information.

**Figure 3:**
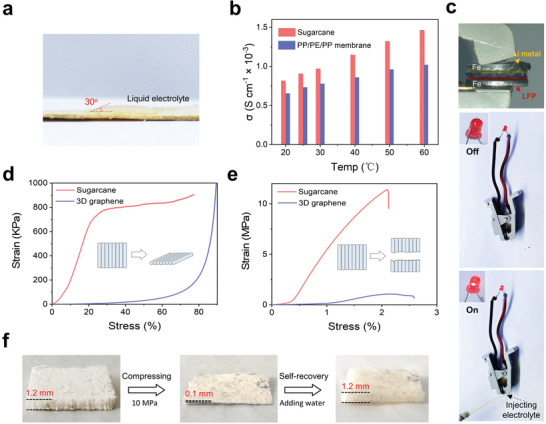
a) Contact angle test when dropping liquid electrolyte (1 m of LiPF_6_ in ethylene carbonate/dimethyl carbonate) on compressed sugarcane film. b) Ionic conductivity of sugarcane and Celgard membrane. c) Unpacked cell running after adding liquid electrolyte from the side edge of cell. Photo of the unpacked cell where dry Sug‐Li||Sug‐LFP (without electrolyte) was sandwiched between two iron sheets (top). A common battery clamp was used to hold the sheel‐less system. After adding liquid electrolyte, dry Sug‐Li||Sug‐LFP ran rapidly. All the operation conducted in argon‐filled glove box. Compression (d) and tensile (e) tests of sugarcane chip and 3D graphene counterpart. 3D graphene was prepared by freeze‐drying of graphene oxide dispersion (50 mg mL^−1^) and the subsequent vapor‐phase reduction process (exposed into hydroiodic acid at 80 °C for 1 h). f) Compressed sugarcane shows self‐recovery behavior after adding water on surface.

Wettability by electrolyte and the corresponding ionic conductivity are of highly importance for our structural‐integrated LMB, especially for the middle sugarcane segment which actually plays the role of separator. We found that sugarcane has good wettability by the common liquid electrolyte with a contact angle of 30^o^ (**Figure** **3**a), which performs better than the common separator (trilayer Celgard 2325 PP/PE/PE membrane) with electrolyte of contact angle of ≈66^o^. ^[^ ^51^ ^]^ The porosity of sugarcane was measured to be 92.5–96.8% based on the displacement method in which water was used for absorption, corresponding to a density of 0.047–0.11 g cm^−3^ (see Section 4). As expected, such strong wettability and high porosity are conducive to sufficient uptake of liquid electrolyte, which was measured to be ≈575%, much higher than ≈135% for PP/PE/PE membrane.

To evaluate Li‐ion conductivity of the electrolyte‐wetted sugarcane, EIS method was employed using iron sheets as two blocking electrodes at temperature window from 20 to 60 °C. Here, the ionic conductivity of pure sugarcane chip is calculated to be 9.02 × 10^−4^ S cm^−1^ at 25 °C, higher than that of the common PP/PE/PE membrane (7.29 × 10^−4^ S cm^−1^, Figure 3b). This should be attributed to its higher porosity uptaking electrolyte and vertically channel facilitating ion transport, while for the common polymer‐based membrane, the inner pores are less ordered and the porosity is only 42%.^[^ ^52^ ^]^ The favorable wettability was observed by the running a dry, unpacked all‐in‐one structured LMB with LFP cathode (noted as Sug‐Li||Sug‐LFP) in argon‐filled glove box, as shown in Figure 3c. The indicator LED turned on immediately and maintained for a while after injecting liquid electrolyte from the side edge, indicating fast liquid electrolyte penetration into cell.

Strength and mechanical stability determine the practicability of sugarcane framework in battery, especially for avoiding short circuit for the structural‐integrated battery. Here, mechanical behavior of sugarcane framework were studied by tensile and compression tests. As a common structural framework or conductive host in electrodes, 3D graphene with density of 0.15 g cm^−3^ was employed for comparison. In Figure 3d, sugarcane chip shows a uniform deformation before its 25% of volume compression, corresponding to a strong compression modulus of 4.2 MPa, far larger than that of 79.8 KPa for 3D graphene counterpart. The good stress tolerance should be attributed to the anisotropic honeycomb structure. Actually, similar structural design has long been used for pressure‐resistant materials, such as honeycomb paper.^[^ ^53^ ^]^ Moreover, a chip with horizontally aligned channels was cut in order to study tensile behavior along the channel direction. As shown in Figure 3e, the resultant chip are mechanically strong with a tensile (Young's) modulus of 0.65 GPa and a tensile strength of 11.3 MPa, while the two values are only 94.9 MPa and 0.88 MPa, respectively, for 3D graphene counterpart.

Furthermore, structural stability was further evaluated by encountering deformation. We found that the sugarcane framework have a structural recovery nature after compression. As shown in Figure 3f, compressed sugarcane chip exhibits a self‐recovery ability by absorbing water. SEM image demonstrates that under 10 cycles of compression and self‐recovery, the honeycomb framework and aligned channels remain, as well as the knots (Figure S17, Supporting Information). This phenomenon should be explained by the large volume swelling after liquid absorption of cellulose in sugarcane,^[^ ^54^ ^]^ implying that the sugarcane framework can be reused after extracting cane juice. Interestingly, this structural rebound behavior can be applied to absorbing active materials. In Figure S10b, Supporting Information, LFP slurry was absorbed spontaneously into compressed sugarcane, along with the volume recovery. As expected, the loading depth was confined to be less than 400 µm due to the preserved framework and knots for blocking, which avoids the possibility of short circuit. From TEM images (Figure S18, Supporting Information), we found that the framework is porous with mesopores, similar to the wood‐derived carbon.^[^ ^21^ ^]^ This suggest that ion can actually transport through the honeycomb walls, which is conducive to the promotion of better battery performance. This result is also agreed by the pore size distribution from nitrogen adsorption/desorption test, where the isothermal shows a hysteresis loop with *P/P_0_* from 0.2 to 0.8 (Figure S19, Supporting Information). Apparently, these nanopores facilitate ion transport through the knot layer but block excessive invasion of cathode particles. Moreover, we demonstrated that the all‐in‐one structured Sug‐Li||Sug‐LFP cell ran even suffering a volume compression of ≈36% by 10 MPa of pressure (Figure S20a, Supporting Information). The stable sugarcane framework hosts both cathode and anode and avoids short circuit. As shown in Figure S20b, Supporting Information, with injection, liquid electrolyte penetrated into cell and the indictor LED turned bright gradually. Here we simulated the ion transport behavior of the all‐in‐one structured Sug‐Li||Sug‐LFP cell after compression. Note that even after large compression where the inclined angle of microchannels reduces to 30^o^, the differential concentration △C for Sug‐Li||Sug‐LFP is lower than that for the traditional three‐layered LMB (Figure S21, Supporting Information). This should be attributed to the continuous, straight ion transport pathways.

**Figure 4:**
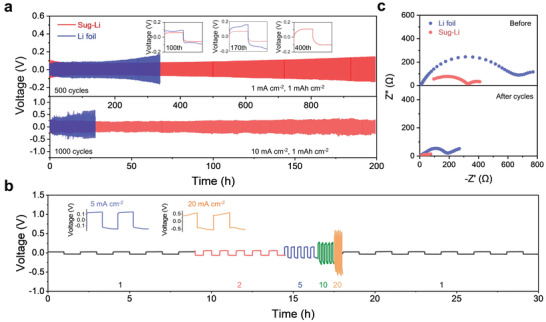
Electrochemical study of Sug‐Li in symmetric cells. a) Voltage‐time profiles of Li stripping/plating cycles of Sug‐Li and Li foil at current densities of 1 mA cm^−2^ and 10 mA cm^−2^. Inset shows enlarged curves at different cycles at 1 mA cm^−2^. b) Rate‐performance of Sug‐Li symmetric cell at current densities ranging from 1 to 20 mA cm^−2^ with stripping/plating capacity of 1 mAh cm^−2^. The right shows the corresponding enlarged cycles at 5 mA cm^−2^ and 20 mA cm^−2^. c) Nyquist plots before and after 100 cycles with current densities of 1 mA cm^−2^ and capacity of 1 mAh cm^−2^.

To evaluate the confined Li metal in sugarcane, symmetric cells were first assembled, where Li metal was loaded on two surface of graphene functionalized sugarcane, noted as Sug‐Li. As shown in **Figure** **4**a, Sug‐Li (areal capacity of ≈10 mAh cm^−2^) exhibits long‐term cycling stability under a stripping/plating capacity of 1 mAh cm^−2^ and current density of 1 mA cm^−2^. Sug‐Li retains a low overpotential of 55 mV after 100 cycles and rises to only 105 mV after 500 cycles. In contrast, Li foil counterpart shows a fast‐growing overpotential from 50 mV at first cycle to 95 mV after 100 cycles. Even short circuit occurs in the 170th cycle, indicating excessive Li metal dendrites impaling separator membrane. The improved cycling stability of Sug‐Li should be attributed to the confined the Li metal in honeycombs. Moreover, such improvement appears to be more obvious at higher current density. In Figure 4b, Sug‐Li runs stable in even 1000 cycle at 10 mA cm^−2^ and keep a less‐fluctuant overpotential of 170–220 mV. While for Li foil, the overpotential rapidly increases to over 500 mV after 100 cycles and results in cell failure soon. Furthermore, we also investigated rate performance of Sug‐Li with current densities from 1 to 20 mA cm^−2^. Likewise, Sug‐Li stays stable during the cycles in which the overpotential is only ≈480 mV for high current density of 20 mA cm^−2^. EIS was also evaluated before and after Li stripping/plating cycles, as shown in Figure 4c. The semicircle in high‐middle frequency region corresponds to the overlapping of interfacial resistance and charge transfer resistance. Obviously, Li metal counterpart has larger interfacial resistance than Sug‐Li for both before and after 100 cycles, suggesting repeated breaking/reestablishing of SEI layer for the “hostless” Li metal. Li plating process of the MnO_2_ functionalized sugarcane kept stable with plating capacity up to 20 mAh cm^−2^, as well as a low overpotential of ≈40 mV (Figure S22, Supporting Information). As expected, “dendrite‐free” morphology in the honeycombs was observed, while Li dendrites are generated on Cu foil with plating capacity of 5 mAh cm^−2^ (Figure S23, Supporting Information). Moreover, graphene‐functionalized sugarcane chip exhibits a high Coulombic efficiency (CE) of ≈96% after 150 cycles under the current density of 1 mA cm^−2^, while the Cu foil counterpart performs far worse in CE test and short‐circuits after 58 cycles (Figure S24, Supporting Information). Moreover, we demonstrated that the structure and morphology of sugarcane remain stable after 1000 cycles, as shown in Figure S25, Supporting Information. The honeycomb wall and knots were shown despite with the advent of wrinkles which may bear stress from cell assembly and electrode volume fluctuation. Likewise, Li dendrites can be hardly observed then (Figure S26, Supporting Information). Also XRD spectrum proved its unchanged structure of cellulose (Figure S27, Supporting Information). We also studied the electrochemical stability of sugarcane under lower and higher temperature. As shown in Figures S28 and S29, Supporting Information, the symmetric cells run stable under both 0 °C and 60 °C, even at high current density of 5 mA cm^−2^ or 10 mA cm^−2^. Also stability at higher temperature was observed by treating sugarcane chip at 150 °C for 2 days (Figure S30, Supporting Information). Moreover, we found that the sugarcane chip maintain integrity and keep good liquid electrolyte absorption ability after cycles, indicating its good structural stability (Figure S31, Supporting Information). The above results indicate that non‐tortuous ion‐transport pathways and increased surface area can lower areal current density and homogenize local Li‐ion flux during stripping/platting.^[^ ^16^, ^19^ ^]^

**Figure 5:**
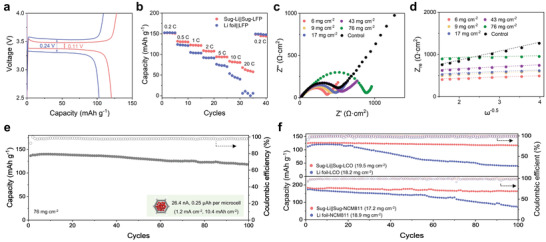
Electrochemical performances of structural‐integrated LMB. Voltage profiles at 1 C (a) and rate capability (b) of Sug‐Li||Sug‐LFP and Li foil||LFP. c) Nyquist plots of Sug‐Li||Sug‐LFP with various loading mass (based on the active material, LFP) and Li foil||LFP counterpart (6 mg cm^−2^). d) Warburg impedance, Z_re_ as a function of *ω* ^−0.5^ for Sug‐Li||Sug‐LFP. e) Cycling stability of Sug‐Li||Sug‐LFP cell at 0.1 C with high loading mass of 76 mg cm^−2^. Inset shows the calculated current and specific capacity per honeycomb column. f) Cycling stability of Sug‐Li||Sug‐LCO and Sug‐Li||Sug‐NCM811 cells.

Next, we evaluated the electrochemical performances of all‐integrated cells by using LFP, LCO and NCM811 as cathodes, noted as Sug‐Li||Sug‐LFP, Sug‐Li||Sug‐LCO and Sug‐Li||Sug‐NCM811, respectively. As shown in **Figure** **5**a, the voltage profiles exhibit that Sug‐Li||Sug‐LFP deliver a higher specific capacity of 122.8 mAh g^−1^ at 1 C than that of 104.9 mAh g^−1^ for Li foil||LFP counterpart. The lower polarization in charging/discharging plateau was observed for Sug‐Li||Sug‐LFP, which agrees with the above conclusion that the honeycomb framework with vertically aligned channels improves electrochemical kinetics in both cathodes and anode. As a result, Sug‐Li||Sug‐LFP behaves significantly improved rate capability in comparison with Li foil||LFP, especially at high current density (Figure 5b). Also the cycling test indicates its long‐term stability of 300 cycles at high current density of 2 C (Figure S32, Supporting Information). We found that the loading mass of cathode materials can be also controlled by changing the amount of slurry in the self‐absorption process (Figure S10, Supporting Information). As shown in Figure S33, Supporting Information, LFP was fully filled into the honeycombs, leaving sugarcane wall and the in‐between gap with width of 2–4 µm after solvent evaporation. Such structure provides direct, continuous ion pathways for fast transport kinetics, also facilitates electrolyte penetration into the inner space of thick cathode.

Typically, the loading mass of LFP can be up to 76 mg cm^−2^, giving a porosity of 60.8% for the whole cell. This may exert high areal capacity matching with Li metal anode. Moreover, Sug‐Li||Sug‐LFP with areal LFP loading mass of 6 mg cm^−2^ exhibits an interfacial resistance of 382 Ω, much smaller than Li foil||LFP counterpart (Figure 5c). And the interfacial resistance of Sug‐Li||Sug‐LFP remains small even when the areal loading mass increases to 76 mg cm^−2^. Moreover, we compared the all‐in‐one structured cell (Sug‐Li||Sug‐LFP) with an assembled cell consisting of Sug‐LFP cathode, Sug‐Li anode and PP/PE/PP membrane (noted as Sug‐Li|PP/PE/PP|Sug‐LFP). As shown in Figure S34, Supporting Information, due to the existence of the common PP/PE/PP membrane, Sug‐Li||Sug‐LFP performs better in rate capacity than Sug‐Li|PP/PE/PP|Sug‐LFP, demonstrating the superiority of all‐in‐one structure.

To further investigate if the integrated structure and favorable wettability translates to good ion‐transport kinetics, Li‐ion diffusion coefficient *D*_Li+_ was calculated by the Warburg impedance in low‐frequency region.^[^ ^16^, ^55^ ^]^ As shown in Figure 5d and Table S1, Supporting Information, Sug‐Li||Sug‐LFP with different loading mass show *D*_Li+_ ranging from 2.7 × 10^−14^ cm^2^ s^−1^ to 1.2 × 10^−13^ cm^2^ s^−1^, which is at least an order of magnitude larger than that of Li foil||LFP counterpart (2.5 × 10^−15^ cm^2^ s^−1^), proving the significant advantage of Sug‐Li||Sug‐LFP in ion transport. Such improvement in Sug‐Li||Sug‐LFP should be attributed to the increased ion‐transport pathways and lowered tortuosity in whole cell. As a result, Sug‐Li||Sug‐LFP cell with high loading mass of 76 mg cm^−2^ runs stable at 0.1 C (1.2 mA cm^−2^) with specific capacity of ≈137 mAh g^−1^, corresponding to mean current value (26.4 nA) and specific capacity (0.25 µAh) per microcell. The corresponding volumetric energy density is calculated to 286 Wh L^−1^. Note that non‐flooded electrolyte was employed for our 3D battery system. Here, the ratio of electrolyte amount to cathode capacity is calculated to be ≈6 µL mAh^−1^, which is a relatively low value due to high cathode loading mass.^[^ ^56^ ^]^ After 100 cycles, it has a capacity retention of 87%. In contrast, Li foil||LFP cell showed a fast decaying capacity after 55 cycles, despite with a lower loading mass of 45 mg cm^−2^ (Figure S35, Supporting Information). Similarly, the improved electrochemical performances were also observed in Sug‐Li||Sug‐LCO and Sug‐Li||Sug‐NCM811 cells. With loading mass over 15 mg cm^−2^, their structural advantage in cycling stability was demonstrated. As shown in Figure 5f, Sug‐Li||Sug‐LCO and Sug‐Li||Sug‐NCM811 show capacity retention of 93.4% and 90.1% at 1 C, respectively, while the capacities for their counterparts, Li foil||LCO and Li foil||NCM811 remain only 33.9% and 41.1% after 100 cycles.

## 3 Conclusion

In summary, we developed a novel strategy on fabricating high‐performance, stable LMBs by integrated structure consisting of paralleled, microcell arrays. Functionalized sugarcane chip was used as framework to host both anode Li metal and cathode material, and creating direct, up and down connected channels in range of whole cell. As a useful framework, sugarcane having honeycomb morphology was proved to have high porosity but also high mechanical strength, as well as the ability of structural self‐recovery. The favorable mechanical properties and sectioned, bamboo‐like microstructure avoids battery short circuit and ensures its stability in practical use. We also demonstrated that the continuous, vertically aligned microchannels greatly facilitate fast, homogenous ion‐transport in both Li anode and cathode, thus suppresses the formation of Li dendrite and improves rate performance and cycling stability of full cells. This is the first demonstration of achieving structural‐integrated LMB with loading cathode and anode in a framework. This strategy provides an architectural model by structural engineering for high‐performance, stable LMB.

## 4 Experimental Section

### Synthesis of LG Sheets

The synthetic processes were performed based on the previous reports.^[^ ^47^, ^48^ ^]^ Typically, graphite flakes (0.1 g, ≈500 µm of flake size) and CrO_3_ (0.85 g) were added into hydrochloric acid (37 wt%, 0.7 mL) slowly. The system was stirred at room temperature for 2 h, giving CrO_3_‐intercalated graphite (GIC). Then GIC was treated by immersing in H_2_O_2_ (10%, 20 mL) for 2 days, forming chemically expanded graphite (CEG) with over 100‐fold volume expansion. Then CEG was oxidized by KMnO_4_‐H_2_SO_4_ system (0.2 g KMnO_4_ in 4 mL concentrated H_2_SO_4_) at 35 °C for 6 hours. The dark‐green mixture was added slowly into ice‐water (50 mL) and then H_2_O_2_ (0.5 mL, 10%) was added to remove the residual oxidant and insoluble substance. Since the resulted oxidized CEG tend to precipitate out from aqueous solution, the residual acid and ions were removed by repeated sedimentation/wash. Then the oxidized CEG was exfoliated by mechanical stirring at 1000 rpm for 10 min, giving large‐sized graphene oxide (LGO) sheets with lateral size of ≈128 µm. In order to obtain conductive large‐sized graphene, LGO was chemically reduced by hydroiodic acid aqueous solution (2%, 1 L). After treated at 90 °C for 4 h, LGO sheets were converted into black, large‐sized graphene sheets (LG, ≈67 µm in mean size). The resultant LG sheets can be re‐dispersed into *N*‐methyl‐2‐pyrrolidone (NMP) or ethanol, form stable dispersion.

### Preparation of Functionalized Sugarcane Chip

Sugarcane was purchased from market and cut into chips for use. The sugarcane chip was washed repeatedly by water and ethanol in order to remove cane sugar and soluble organics. In order to enhance the conductivity, two surfaces of sugarcane chip were functionalized by LG sheets. Here, LG‐NMP dispersion (1 mL, 1.2 mg mL^−1^) was added drop‐wise onto the surfaces of dry sugarcane chip (≈1.2 cm in thickness and ≈3 cm^2^ in surface area). Due to the knot‐sectioned structure of sugarcane, LG sheets were filtered onto the surface area with functionalized depth of ≈300 µm, thereby avoiding excessive functionalization of whole chip. Furthermore, in order to improve the Li metal wettability of sugarcane, one surface of sugarcane chip was functionalized by MnO_2_ nanoparticles. Similarly, MnO_2_ nanoparticles were dispersed into ethanol by ultrasonic treatment for 2 h, form MnO_2_ dispersion with concentration of 1 mg mL^−1^. Then the dispersion was added drop‐wise onto one surface of the resultant sugarcane chip, giving black surface. After vacuum drying at 60 °C for 5 h, LG‐MnO_2_ functionalized sugarcane chip was obtained.

### Preparation of All‐in‐One Structured LMB

Cathode was first loaded into the resultant chip from graphene‐functionalized surface by slurry coating or a self‐absorption process. Here, cathode slurry was prepared by dispersing commercial cathode powder (for example, LFP), super P and polyvinylidene fluoride (with weight ratio of 8:1:1) in NMP. The resultant cathode slurry was then coated onto the chip (for slurry coating method) or compressed chip (for self‐absorption method) from the graphene‐functionalized surface. After standing for 2 h for complete absorption, the cathode loaded chip was putted onto a collector Al foil with touched by the cathode side. Then the chip was vacuum‐dried at 80 °C for 10 h and the Al foil was pasted, as shown in Figure S1, Supporting Information. The areal loading mass of cathode materials can be controlled by the amount of cathode slurry. For self‐absorption method, the volume of compressed chip swelled during slurry absorption, therefore enables high areal loading mass. Next, lithium metal was absorbed into the cathode‐loaded chip from the MnO_2_‐loaded surface. Here, in argon‐filled glove box with oxygen and water content below 1 ppm, Li foil with certain thickness was placed onto iron plate and cathode‐loaded chip was put onto the Li foil touching the MnO_2_‐loaded surface. Then after heating at 250 °C for 5 min, lithium was absorbed into the honeycomb channels, showing metallic luster. Likewise, the loading mass of lithium metal can be decided by the thickness of Li foil. Cu foil was covered onto Li metal anode as collector. Here the anode and cathode capacity ratio was set to be around 3:1. Finally, the resultant cathode‐Sug‐Li metal chip was punched into discs for electrochemical characterizations.

### Electrochemical Simulation

Electrochemical simulation was conducted using COMSOL Multiphysics software.^[^ ^43^ ^]^ For the all‐in‐one structure with vertically aligned channels, the width and wall thickness were set as 5 µm and 50 µm respectively, according to SEM image of sugarcane honeycombs. LFP cathode with porosity of 30% was filled into the honeycombs for simulation. While for the traditional three‐layered Li metal battery, membrane layer (50 µm in thickness) with porosity of 42% was applied for separator, combining with LFP cathode and Li metal. In the initial stage, all the channels/pores in two structures were filled by 1 m of electrolyte. The simulations were proceeded with current density of 1 mA cm^−2^ and discharging window ranging from 4.0 V to 2.5 V. Real‐time Li‐ion concentration was finally calculated to characterize Li‐ion transport kinetics in the two structures.

### Electrolyte Uptake and Porosity Measurement

Typically, sugarcane chip was immersed in water for 2 h for fully absorption. Then the absorbed water on surface was removed. The electrolyte uptake (*U*) and porosity can be calculated as

(1) $$U\;=\frac{m_{2}\;-\;m_{1}}{\;m_{1}}\;\times \;100\%$$

(2) $$\mathrm{Porosity}=\frac{m_{4}-m_{3}/\rho_{\mathrm{water}}}{V_{\mathrm{Sugarcane}}}\;\times \;100\%$$

where *m_1_* and *m_2_* are the weight of sugarcane before and after absorbing electrolyte respectively, *m_3_* and *m_4_* are the weight of sugarcane before and after absorbing water respectively, *ρ*_water_ is the density of water (1 g cm^−3^) and *V*_Sugarcane_ is the apparent volume of sugarcane chip.

### Electrochemical Analysis

Electrochemical measurements of symmetric cells and Li metal cells were carried out using CR‐2032 coin cells, operated in argon‐filled glove box. LiPF_6_ (1 m) in ethylene carbonate (EC)/dimethyl carbonate (DMC) liquid was used as electrolyte. The electrolyte volume was controlled below 80 µL for all cells. For the traditional three‐layered Li metal battery counterparts, Celgard 2325 (PP/PE/PP) membrane was used as the separator. Galvanostatic charge/discharge and Li stripping/platting cycles were conducted in LAND CT2001A multichannel battery testing system. Electrochemical impedance spectroscopy (EIS) were tested using BioLogic VMP‐300 electrochemical system at open circuit potential in frequency window of 1 MHz–100 mHz at an amplitude of 10 mV.

### Calculation of Li‐Ion Diffusion

Li‐ion diffusion coefficient (*D*_Li+_) was calculated by the Warburg impedance in low‐frequency region, expressed as:

(3) $$D_{Li^{+}}=\frac{R^{2}T^{2}}{2A^{2}n^{4}F^{4}C^{2}\sigma^{2}}$$

where *R* is gas constant, *T* is absolute temperature, *A* is the surface area of electrode, *n* is number of electrons per molecule during electrochemical reaction, *F* is Faraday's constant, *C* is concentration of Li^+^ (0.0227 mol cm^−3^ for LFP), and *σ* is Warburg factor.^[^ ^55^ ^]^ Here *σ* can be obtained according to the line slop of *Z_re_* versus *ω* ^−1/2^.

### Equipment

The morphology and structure of materials were characterized by SEM (JSM‐7800F Prime), TEM (Tecnai G2 20 TWIN and JEM‐2100F, 200 kV), XTM (ZEISS Xradia 510 Versa), Mechanical properties (INSTRON 5966 system), XPS (ThermoFisher Scientific, ESCALAB 250Xi), XRD (Bruker D8 Advance, operated at 40 mA), BET (Quantachrome Autosorb‐iQ‐MP‐AG) and Optical microscopy (Leica DM2500P).

## Conflict of Interest

The authors declare no conflict of interest.

## Supporting information

Supporting Information

## Data Availability

The data that support the findings of this study are available in the supplementary material of this article.
