# Supplementary material for: All‐in‐One Structured Lithium‐Metal Battery
Source: Adv Sci (Weinh). 2022 Apr 13;9(17):2200547. doi: 10.1002/advs.202200547 (PMC9189645; doi:10.1002/advs.202200547)
Supplement: Supplementary file 1 — Supporting Information [file ADVS-9-2200547-s001.pdf]

ADVANCED SCIENCE
Open Access

Supporting Information

for *Adv. Sci.*, DOI 10.1002/advs.202200547

All-in-One Structured Lithium-Metal Battery

*Lei Dong, Chang Zhang and Wei Liu**

Supporting Information

All-in-one structured lithium-metal battery

Lei Dong[1], *Chang Zhang*[1], *Wei Liu*[1*]

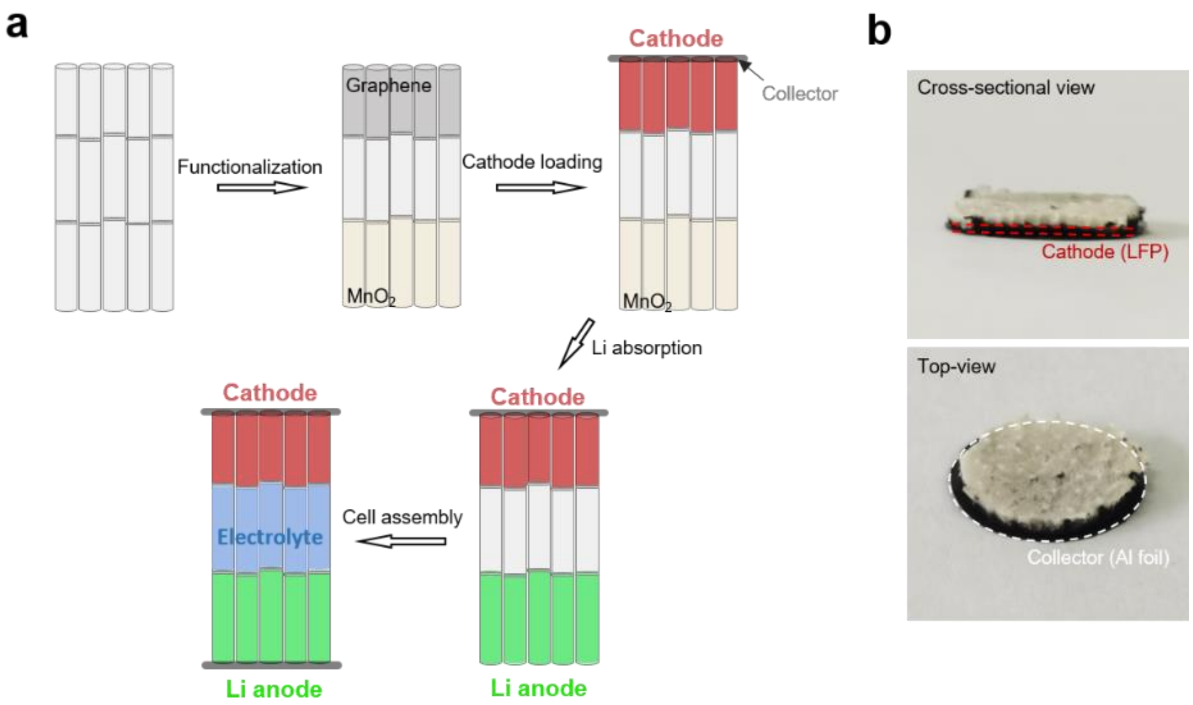

Figure S1. (**a**) Fabrication processes of our structural-integrated Li-metal battery (LMB). Cathodic materials and Li metal were loaded from the top and bottom surface of functionalized sugarcane, respectively. (**b**) Photos of cathode (LFP)-loaded sugarcane chip with collector. Here the fresh, wet cathode-loaded sugarcane chip was posted onto collector Al foil. Because of the contained binder in cathode slurry, Al foil has close contact with the cathode-loaded sugarcane chip after drying.

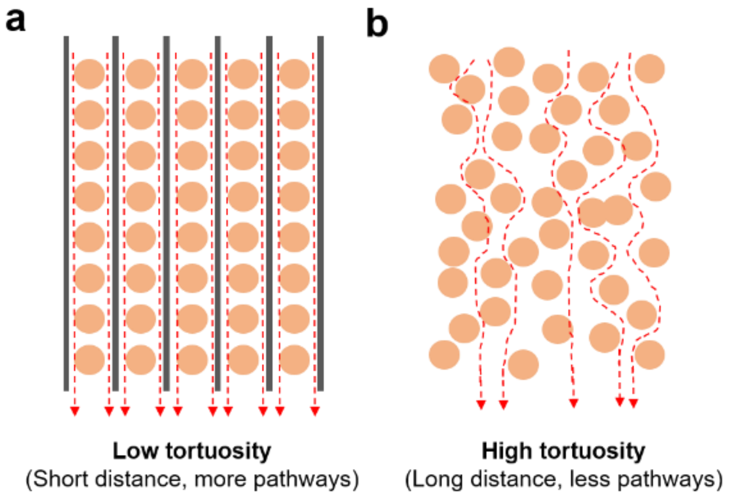

Figure S2. Schematic illustration of vertical-structured electrode and the common disorder-structured electrode.

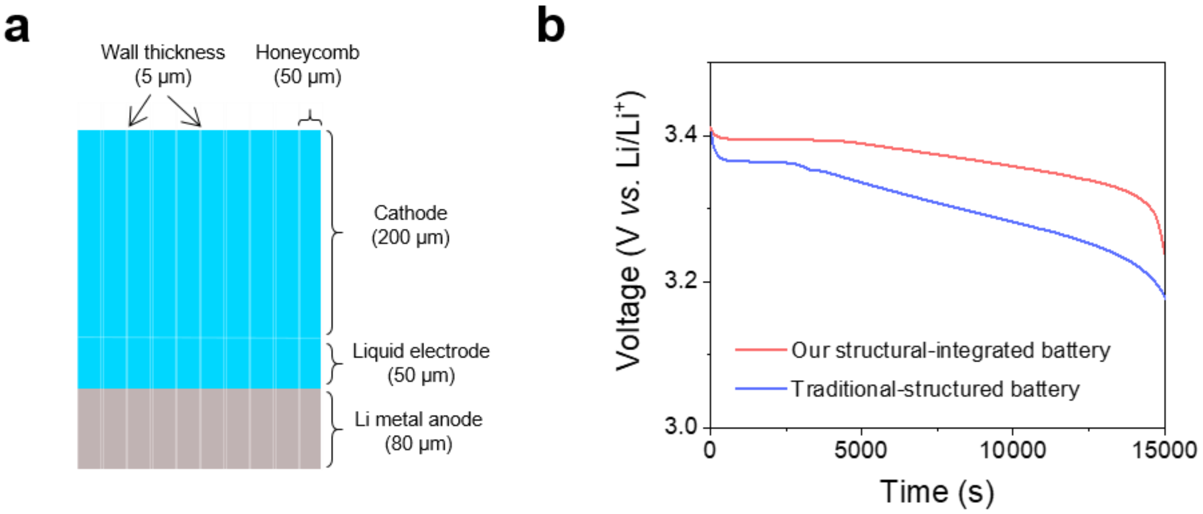

Figure S3. (**a**) Structural model of the structural-integrated Li-metal battery for simulation. Initial Li-ion concentration is set to be 1 M and the LFP-based batteries are discharged at current density of 1 mA cm^{-2}. (**b**)The simulated voltage response as function of time during the discharging process.

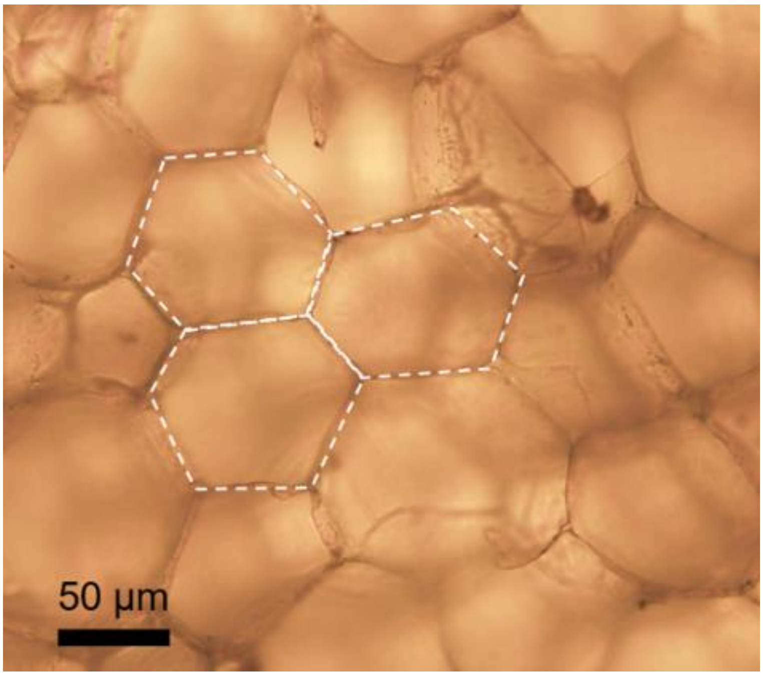

Figure S4. Optical image of sugarcane shows honeycomb structure.

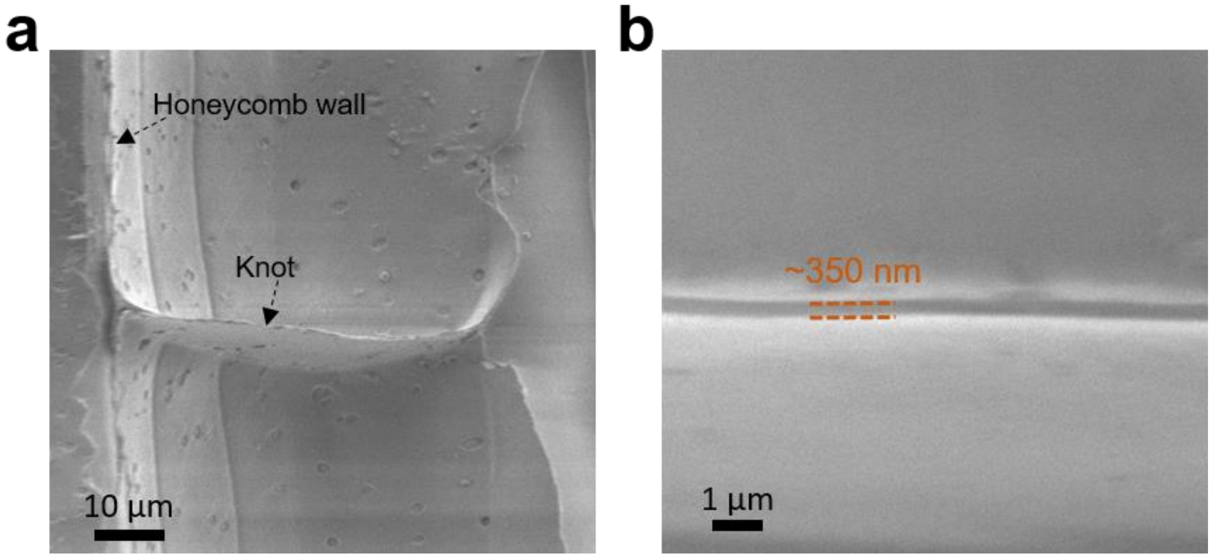

Figure S5. (a) Cross-sectional SEM image of sugarcane. (b) Enlarged SEM image at knot area.

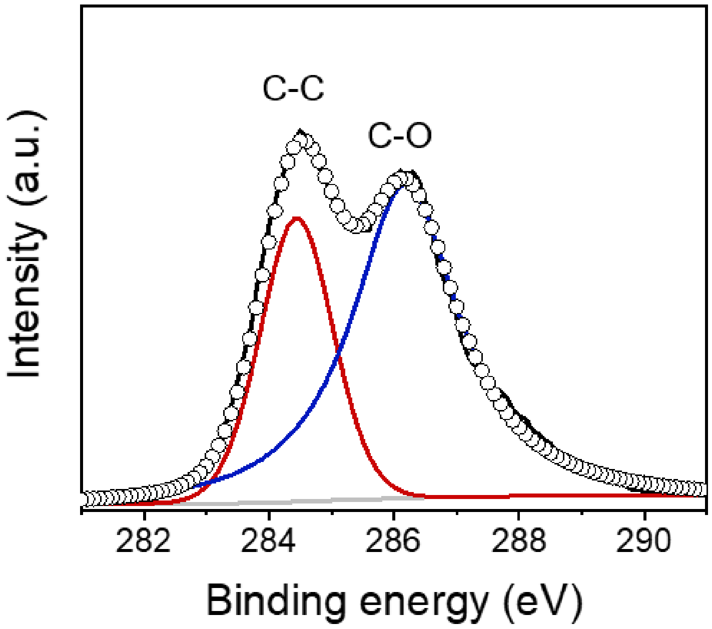

Figure S6. XPS C spectra of sugarcane after washed by water and ethanol. The functional groups of C-C and C-O bonds agree with its componet of cellulose[4,5].

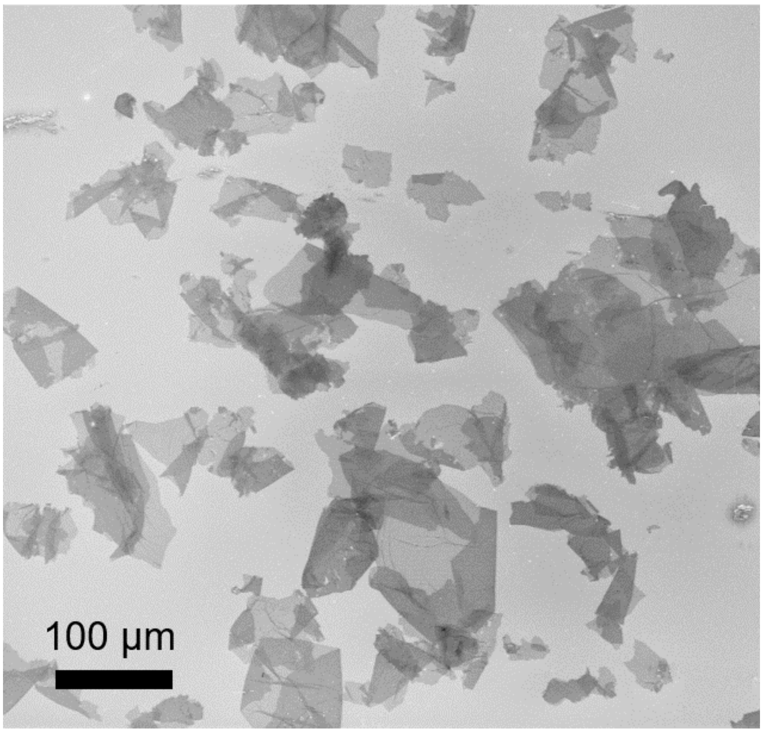

Figure S7. SEM image of large-sized graphene oxide prepared according to our previous report.[3]

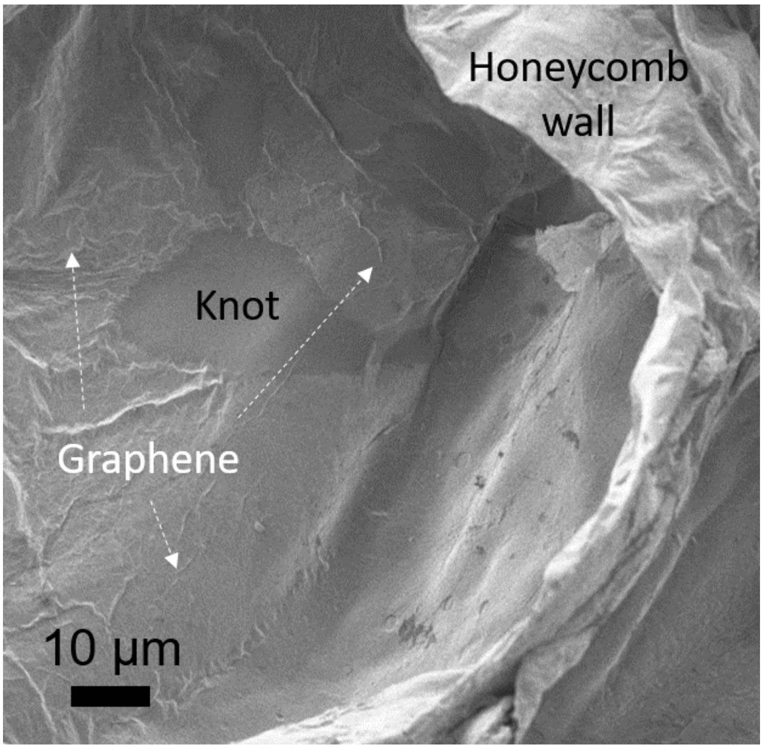

Figure S8. SEM image of large-sized graphene functionalized sugarcane where the wrinkled graphene sheet, honeycomb wall and knot can be well distinguished. Here, graphene sheets were filtered and blocked by the knots in the microchannel in sugarcane.

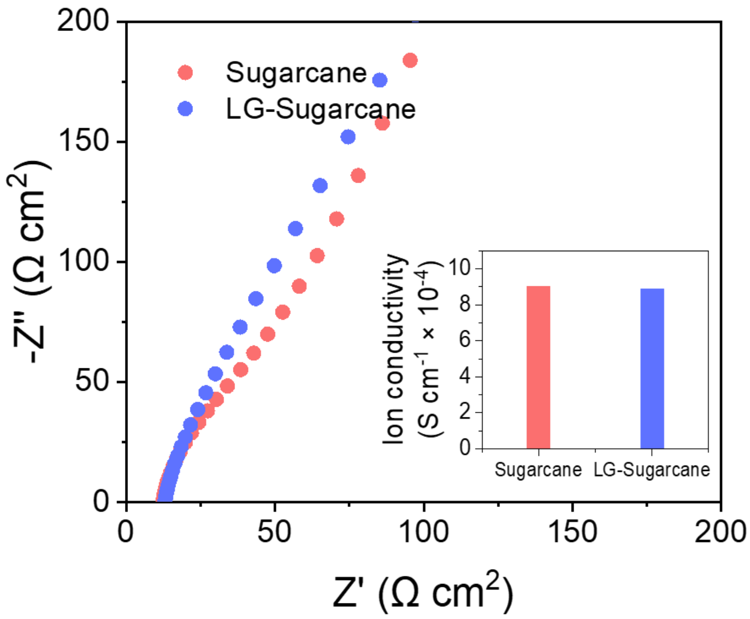

Figure S9. Nyquist plots of sugarcane and LG-functionalized sugarcane (LG-Sugarcane). The thickness of sugarcane chips is around 400 μm. The cells were assembled by sandwiching sugarcane chips between two iron slice. Sugarcane was wetted by electrolyte (1 M of $LiPF_6$ in EC/DEC) before test. Inset shows the corresponding ion conductivities.

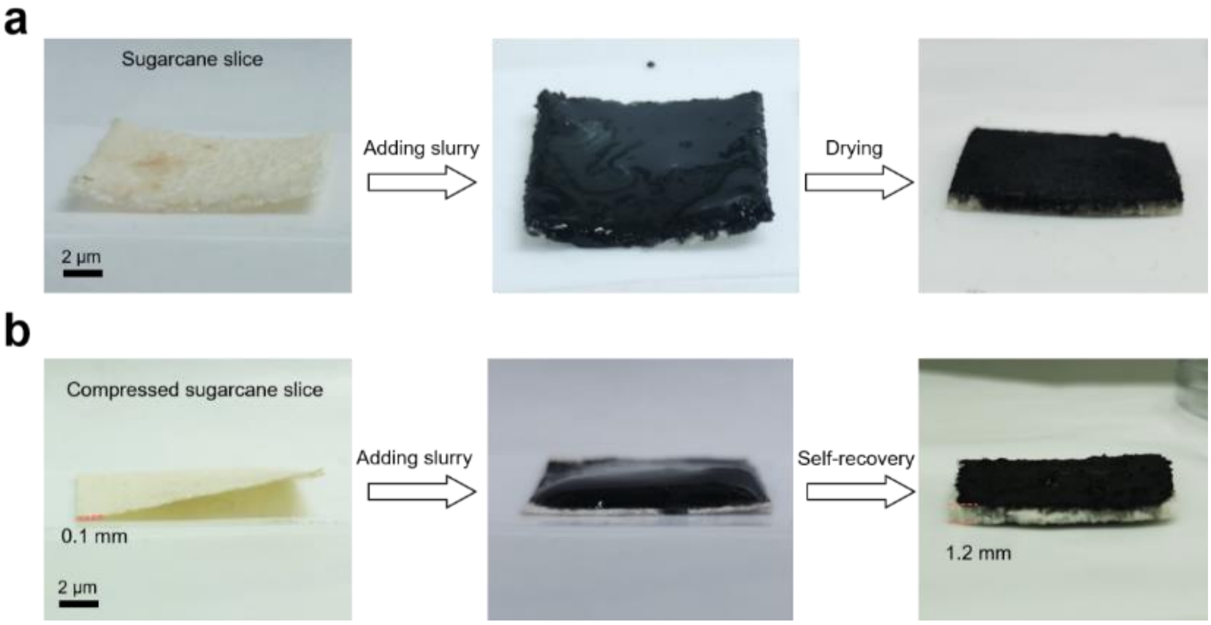

Figure S10. (**a,b**) $LiFePO_4$ (LFP) slurry was loaded into sugarcane chip by coating (a) or self-absorption (b) methods. When slurry was added onto the surface of compressed sugarcane chip, slurry was absorbed slowly into sugarcane, along with volume self-recovery of sugarcane chip.

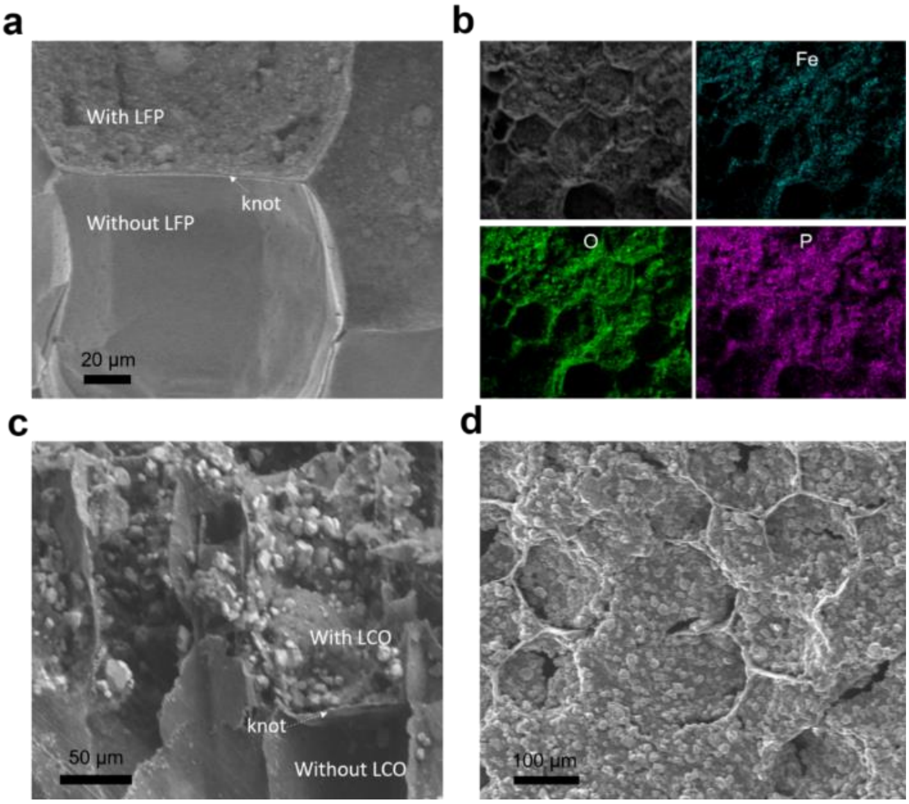

Figure S11. (**a,b**) SEM image and elemental mappings of sugarcane chip loaded by LFP. Top-left in b shows the corresponding SEM images for elemental mappings. (**c,d**) SEM

images of sugarcane chip loaded by $LiCoO_2$ (LCO). The active materials are well confined by sugarcane honeycomb and the knots.

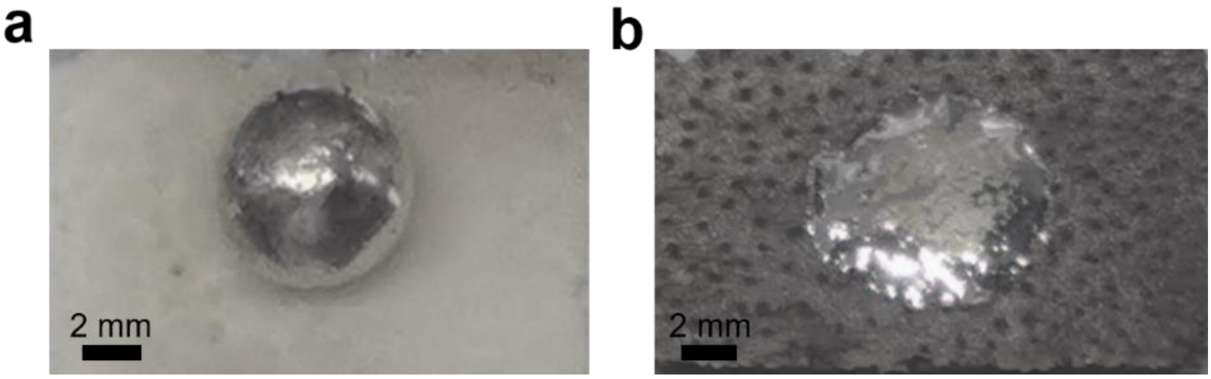

Figure S12. (**a,b**) Optical images shows the wettability of raw sugarcane (a) and MnO_2-functionalized sugarcane (b) by adding molten lithium metal on surface.

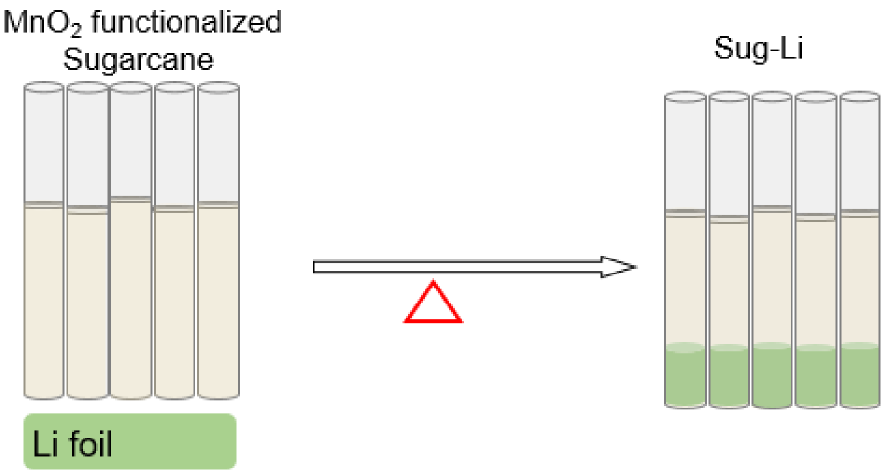

Figure S13. Melton lithium metal was absorbed into the MnO_2 functionalized sugarcane. The loading depth (thickness) of Li metal depends on raw Li foil usage amount and is restricted in MnO_2 functionalized region due to the lithiophobic nature of sugarcane.

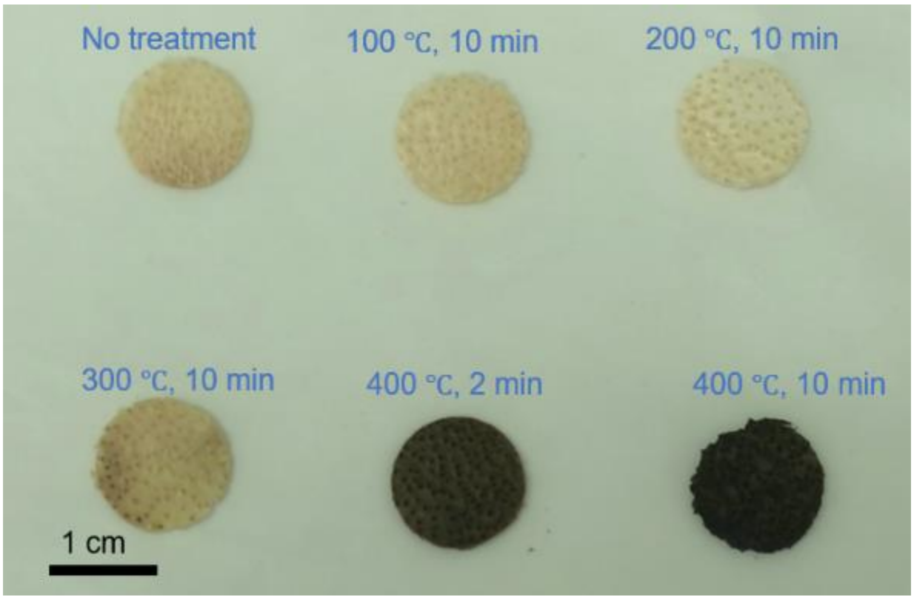

Figure S14. Photos of sugarcane chip after heat treatment (different temperature and time).

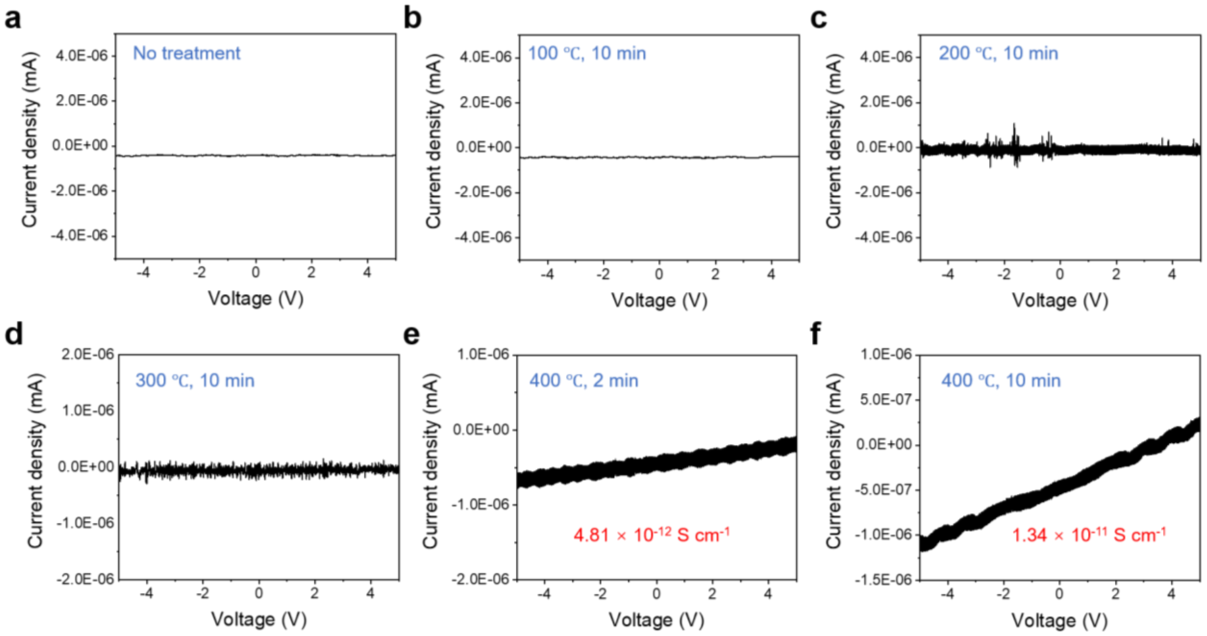

Figure S15. Current-Voltage (I-V) curves of dry sugarcane chip after different heat treatment. The current change is negligible for a-d because of the insulated nature of sugarcane. After treatment at 400 °C, the electrical conductivities are calculated to be 4.81×10^{-12} S cm^{-1} for e and 1.34×10^{-11} S cm^{-1} for f. Note that these values are quite low and then the sugarcane should also be considered as insulator.

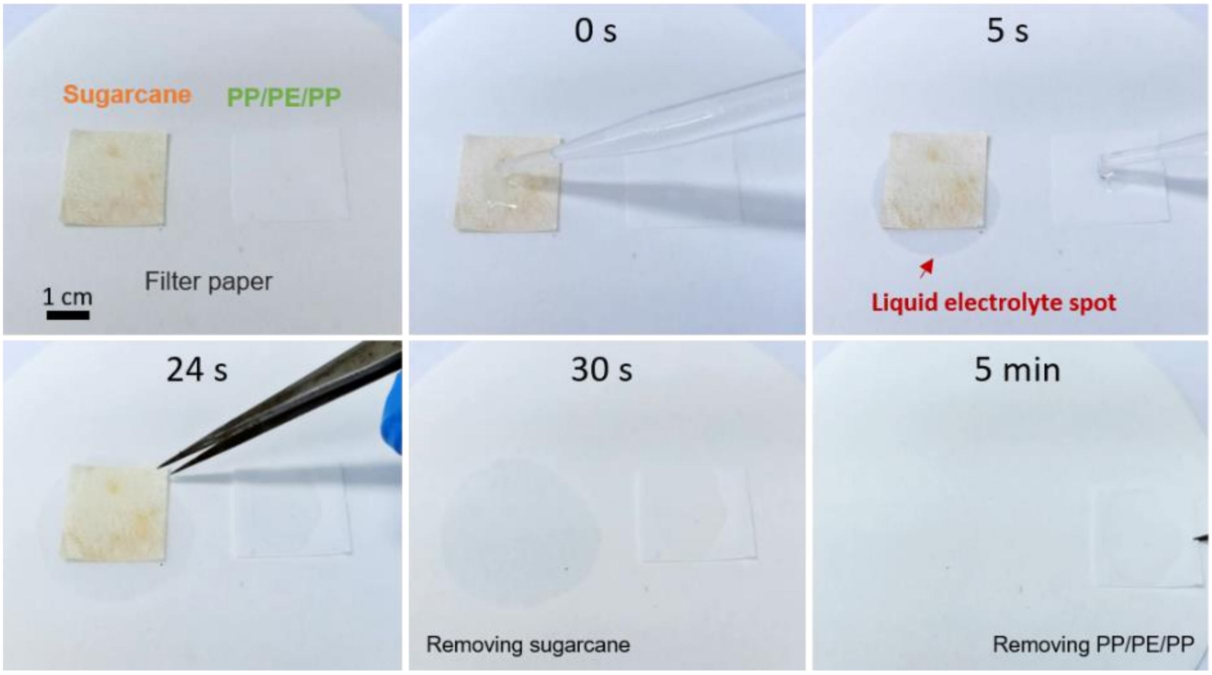

Figure S16. Liquid electrolyte (1 M of $LiPF_6$ in EC/DEC) rapidly penetrated sugarcane film and form a liquid spot on the underneath filter paper in 5 s. In contrast, it is relatively difficult to through PP/PE/PP membrane.

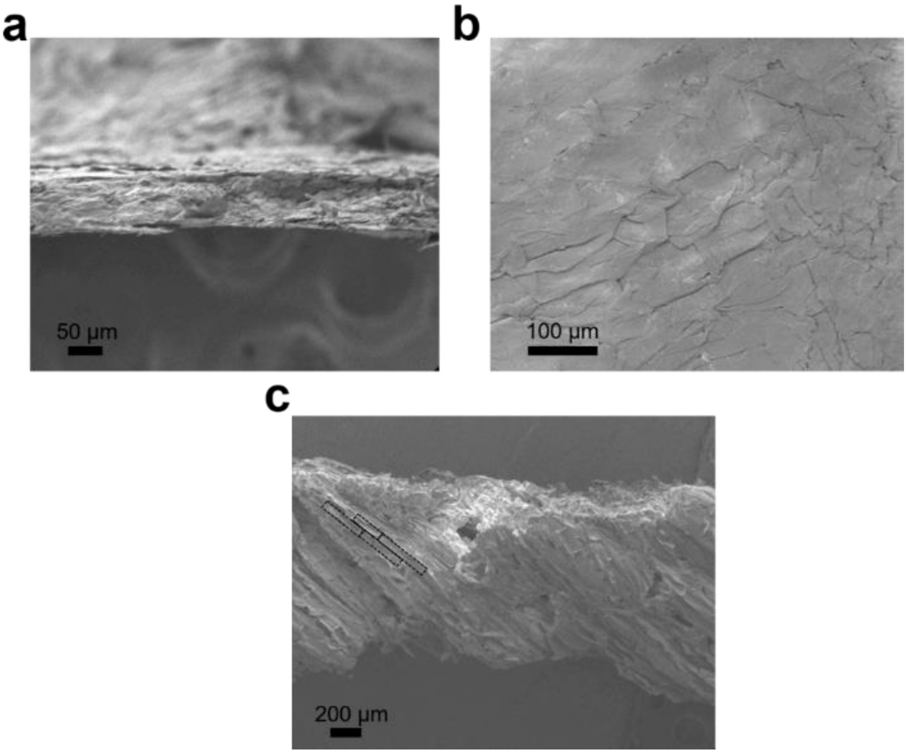

Figure S17. (**a-c**) SEM image of compressed sugarcane (a) and after 10 cycles of compression-self recovery (c). Despite small structure distortion, the sectioned microchannels and knots can still be distinguished in c.

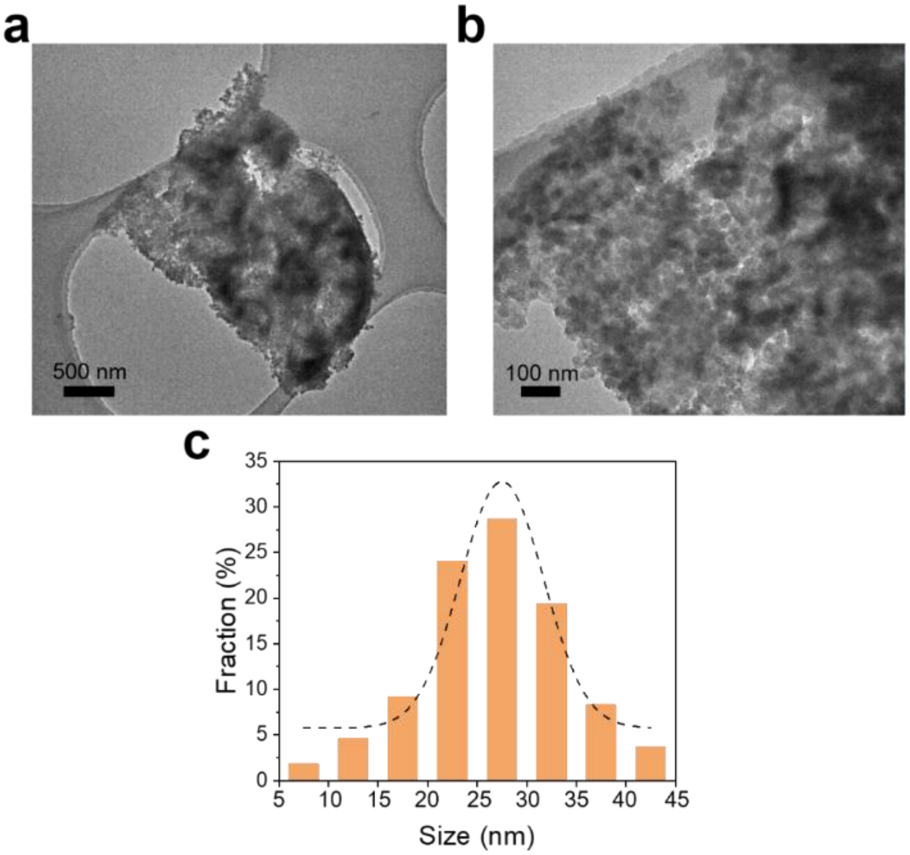

Figure S18. (**a,b**) TEM images of exfoliated sugarcane showing porous structure. (**c**) Size histograms from b after counting for over 100 cellulose nanoparticles.

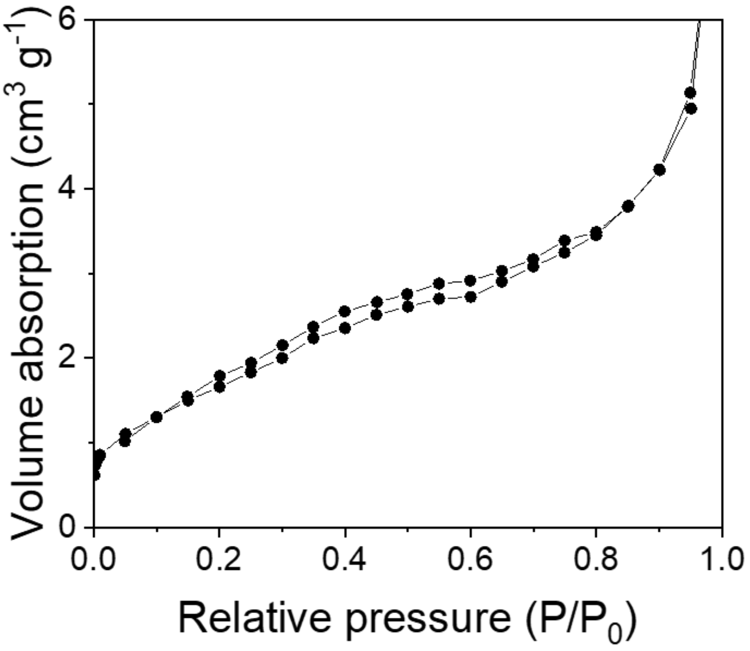

Figure S19. Volume absorption-relative pressure curve by nitrogen adsorption/desorption test.

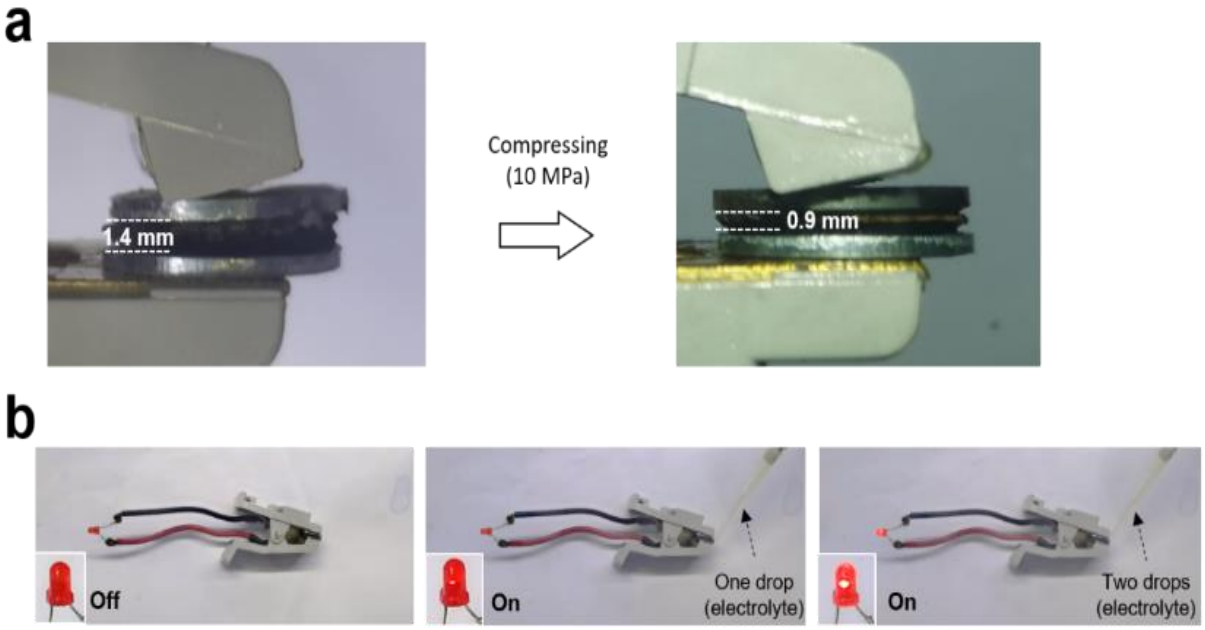

Figure S20. (**a**) Unpacked cell was largely compressed by 10 MPa of pressure. (**b**) After adding one drop of liquid electrolyte, compressed Sug-Li||Sug-LFP cell ran rapidly. The indicator LED appeared brighter after adding the second drop. All the operation conducted in argon-filled glove box.

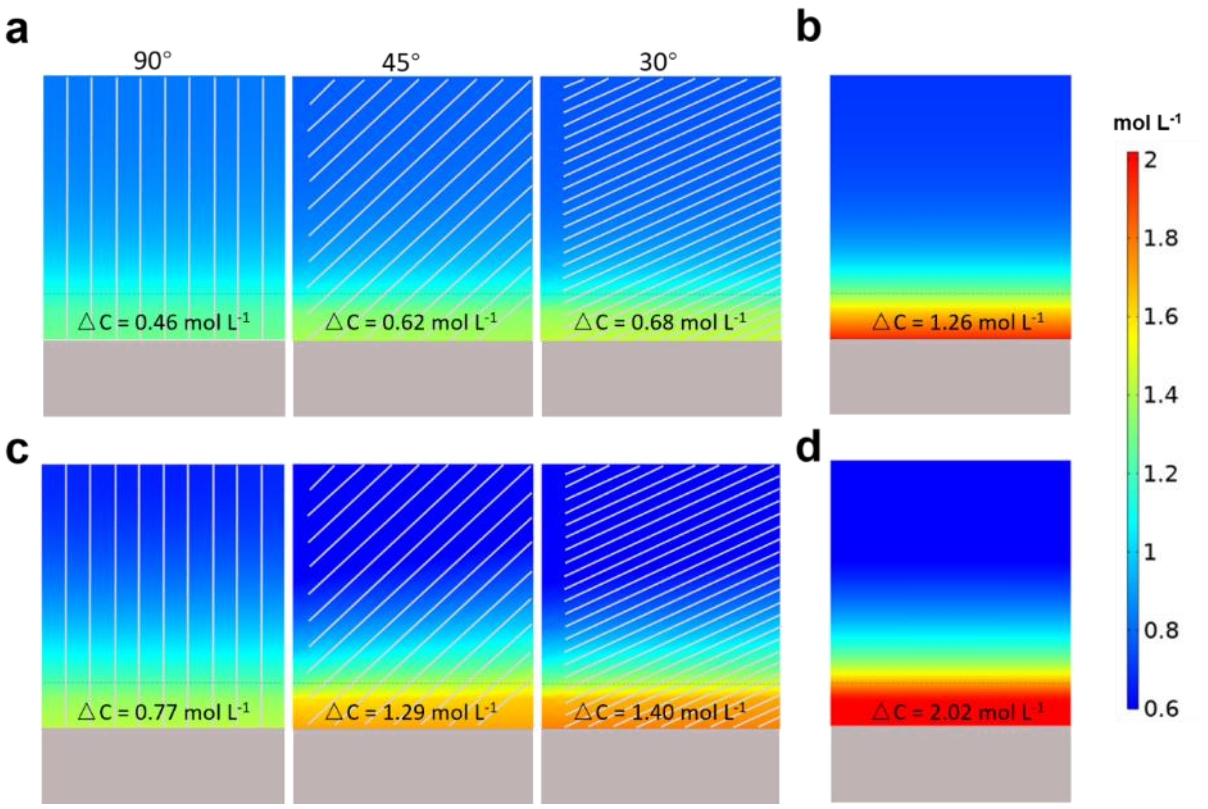

Figure S21. (a-d) Simulation of ion-transport kinetics in compressed all-in-one structured lithium metal battery (a,c) and traditional lithium metal battery with disordered porous microstructure (b,d). Color mappings represent local ion concentration at different reaction time of 6000 s (a,b) and 15000 s (c,d). Inset in *a* and *c* show the corresponding inclined sugarcane wall with inclined angle from 90° to 30°. The simulations were proceeded with current density of 1 mA cm^{-2} and discharging window ranging from 4.0 V to 2.5 V.

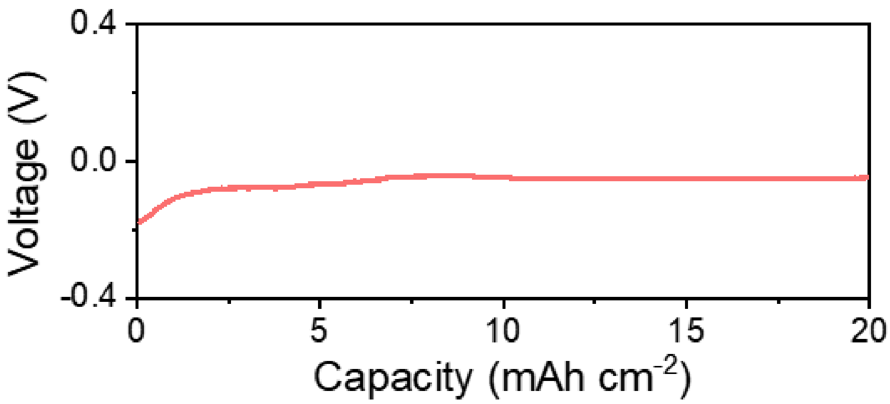

Figure S22. Li plating curve of MnO_2 functionalized sugarcane at current density of 0.5 mA cm^{-2}.

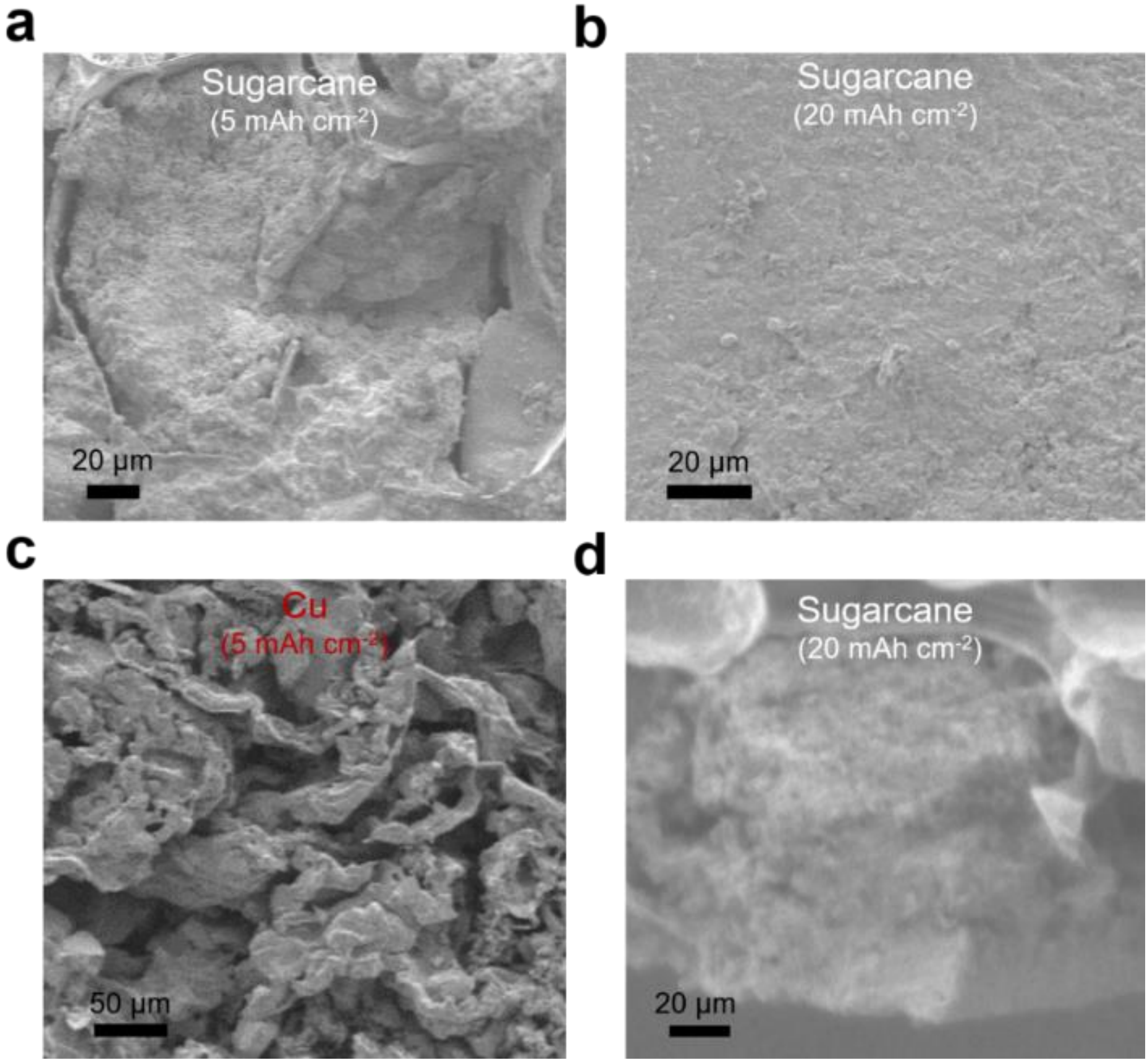

Figure S23. (**a-d**) SEM images shows Li deposition on sugarcane chip (a,b,d) and Cu foil (c) at current density of 0.5 mA cm^{-2}. Apparently, Li metal was well plated into the honeycomb of sugarcane (a) and Li dendrites cannot be observed even with plating capacity up to 20 mAh cm^{-2}. In contrast, Li dendrites were visible for Cu foil.

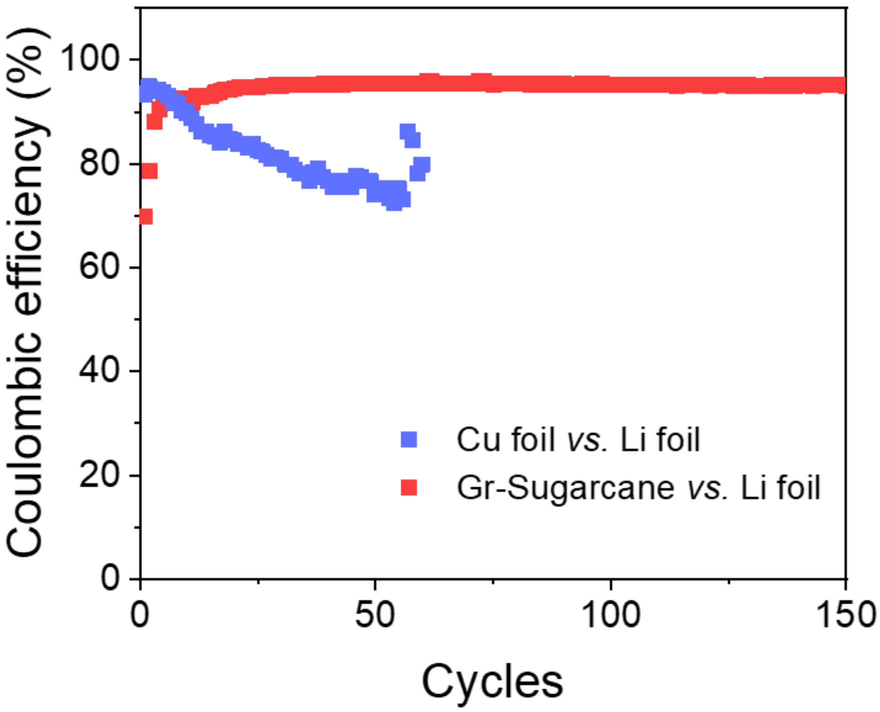

Figure S24. Coulombic efficiency (CE) of Li stripping/platting cycles of graphene-functionalized sugarcane (Gr-Sugarcane) with Cu foil at current density of 1 mA cm^{-2} with a capacity of 1 mAh cm^{-2}.

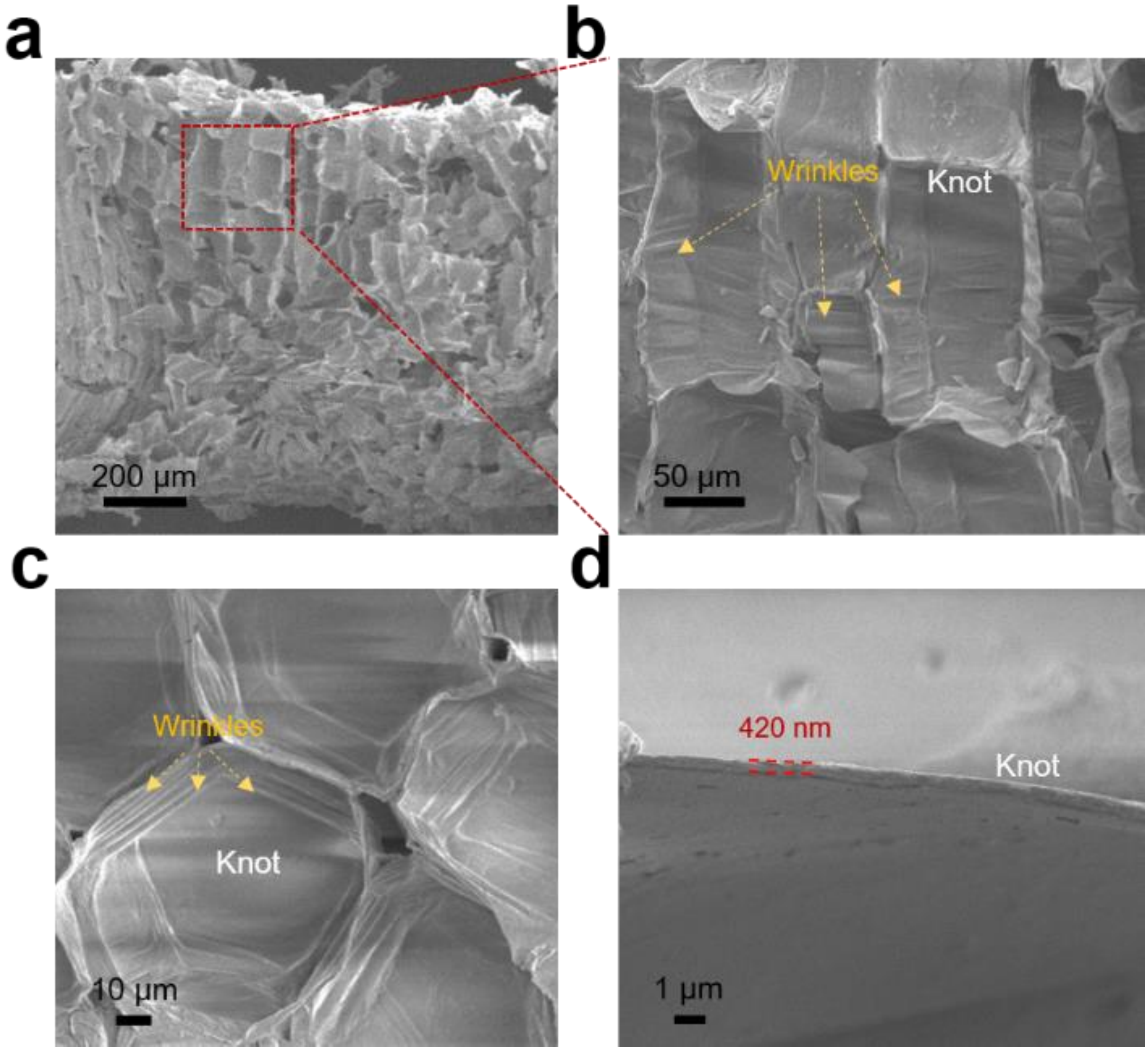

Figure S25. SEM images of sugarcane after 1000 cycles. (**a-c**) Cross-sectional and top-view images shows the remained honeycomb structure with knots. The wrinkles bear stress from cell assembly or electrode volume fluctuation, improving mechanical stability. (**d**) Enlarged image of knot. Here, MnO_2-functionalized sugarcane chip was used as a separator in two Li metal electrodes and ran at current density of 10 mA cm^{-2} and capacity of 1 mAh cm^{-2} for 1000 cycles.

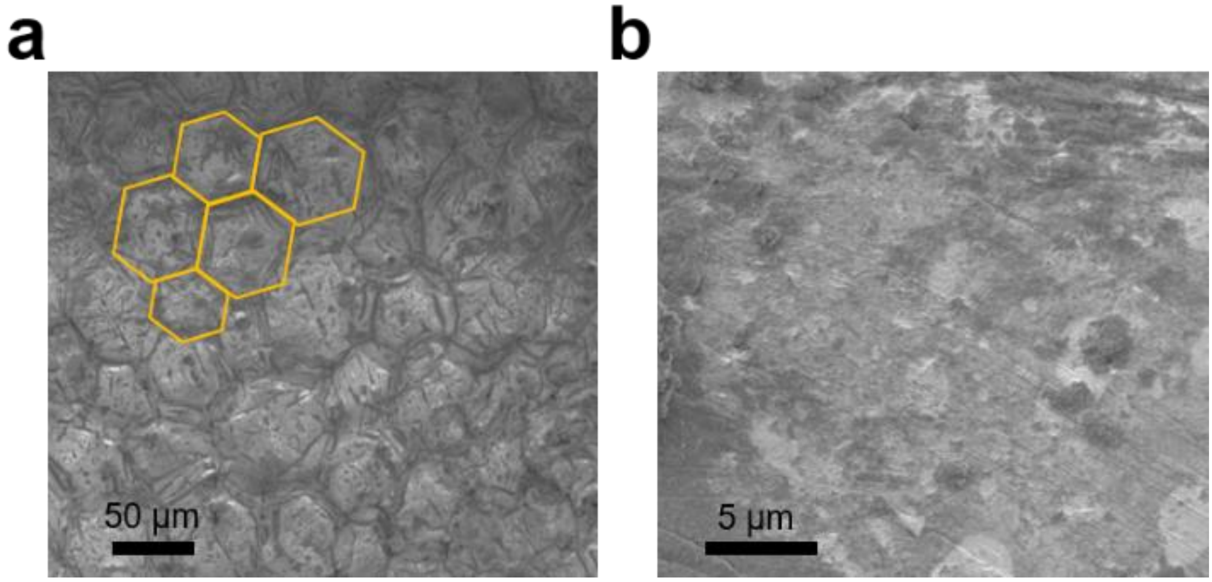

Figure S26. (**a,b**) Top-view SEM image of Li metal in a symmetric battery where MnO_2-functionalized sugarcane chip was used as a separator between two Li metal electrodes and ran at current density of 10 mA cm^{-2} and capacity of 1 mAh cm^{-2} for 1000 cycles. The left honeycomb mark from sugarcane is shown in a and Li dendrites can be hardly observed in b.

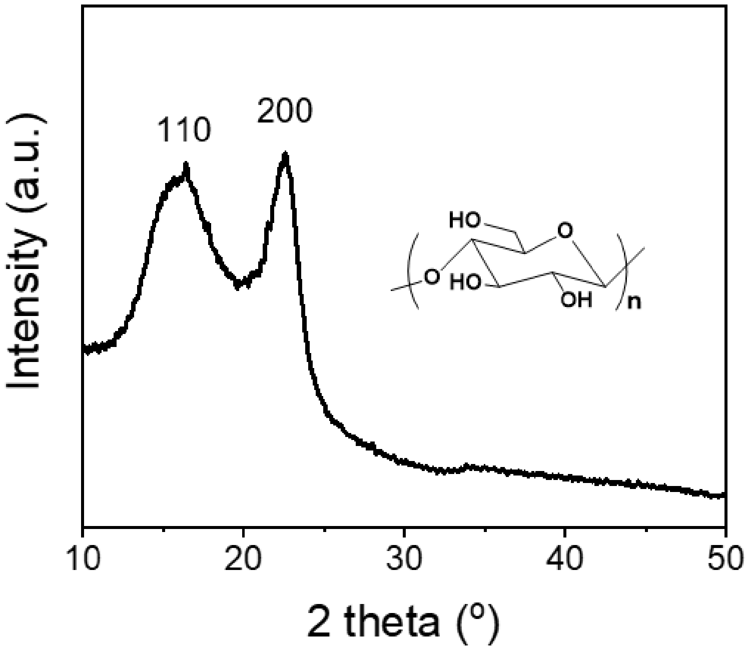

Figure S27. XRD spectra of sugarcane after 100 cycles in Li symmetric battery with current density of 1 mA cm^{-2} and capacity of 1 mAh cm^{-2}.

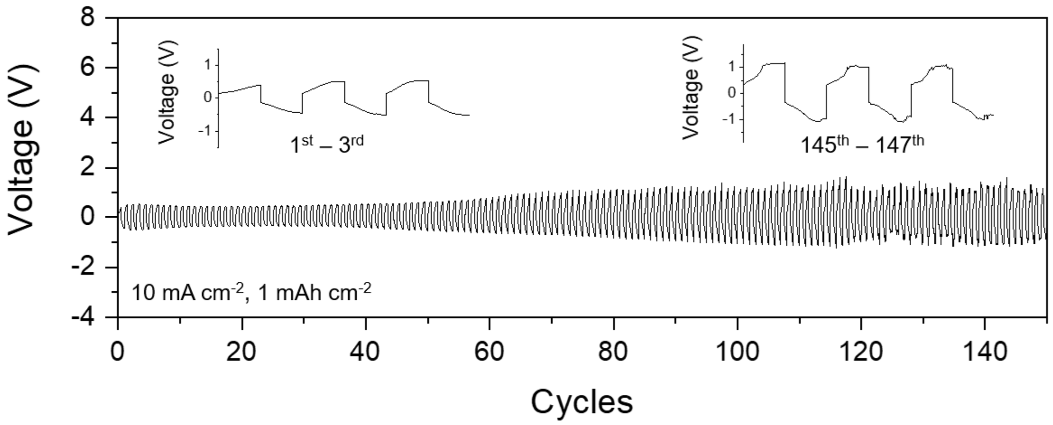

Figure S28. Voltage-time profiles of Li stripping/plating cycles of functionalized sugarcane under a high temperature of 60 ℃ at current densities of 10 mA cm^{-2}. Here, functionalized sugarcane chips were used as separator and liquid electrolyte (1 M of $LiPF_6$ in ethylene carbonate/dimethyl carbonate) were added.

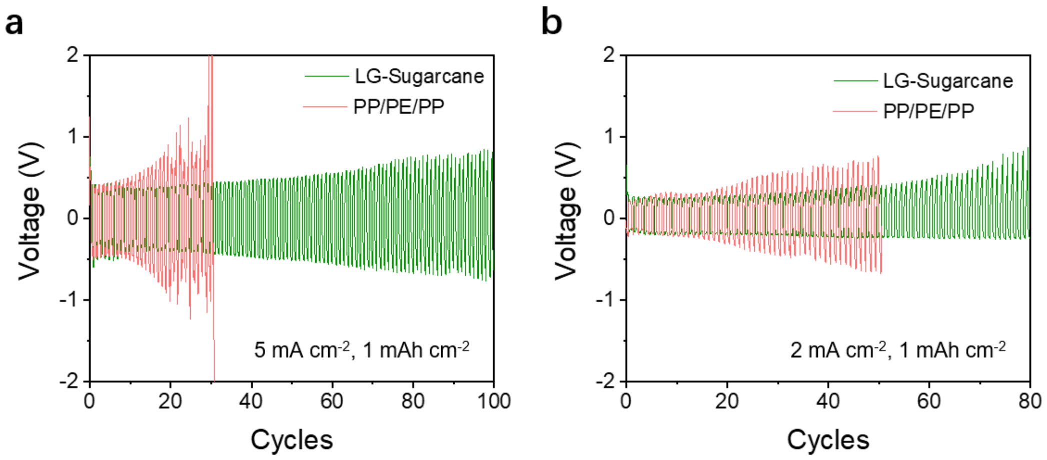

Figure S29. (a,b) Voltage-time profiles of Li stripping/plating cycles of functionalized sugarcane under a low temperature of 0 ℃ at current densities of 5 mA cm^{-2} (a) and 2 mA cm^{-2} (b). Here, functionalized sugarcane chips were used as separator and liquid electrolyte (1 M of $LiPF_6$ in ethylene carbonate/dimethyl carbonate) were added.

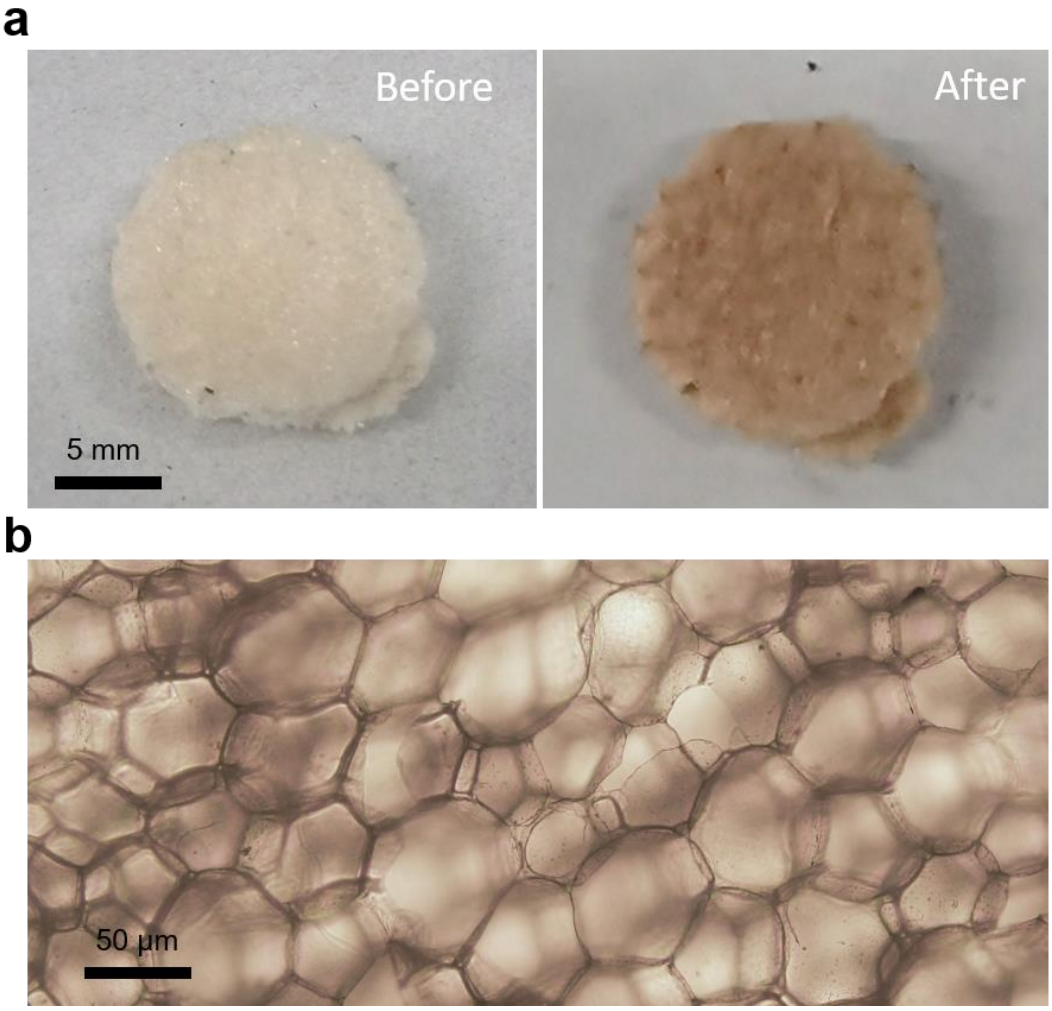

Figure S30. (a,b) Photo (a) and optical image (b) of sugarcane chip before and after standing at 150 ℃ for 2 days.

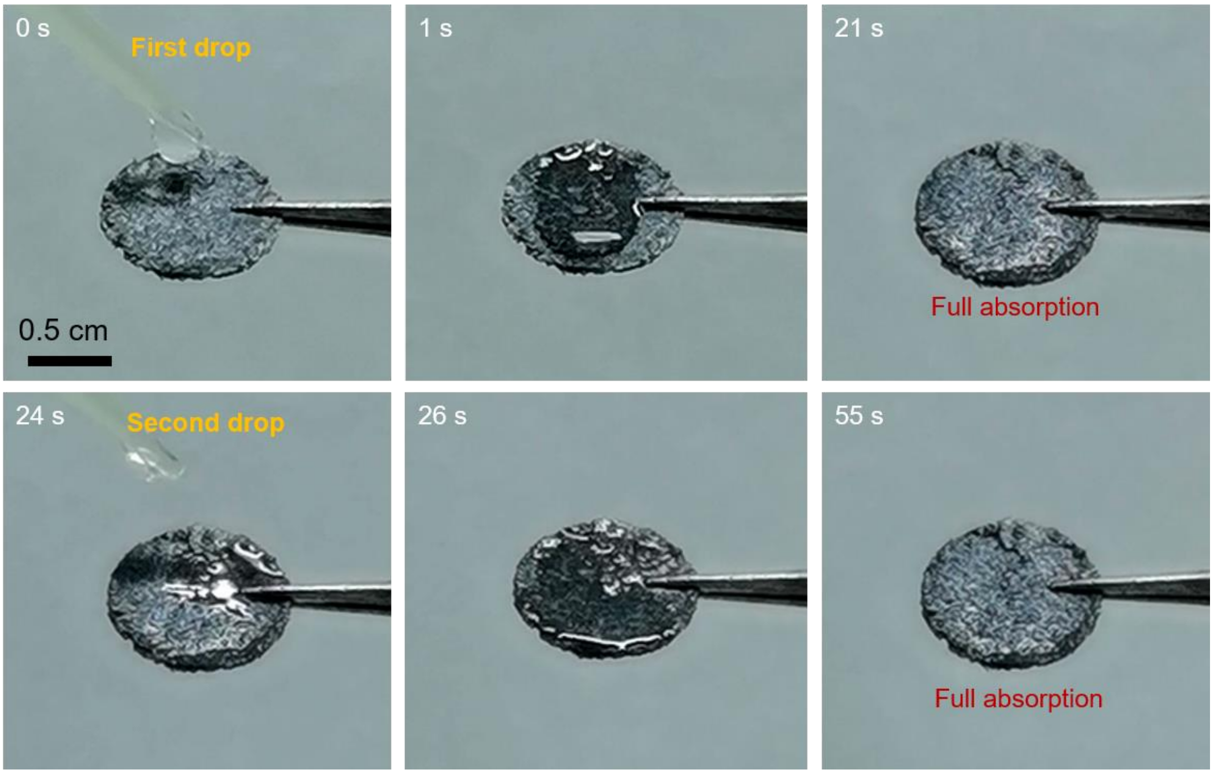

Figure S31. After 100 cycles at current density of 10 mA cm^{-2} and capacity of 1 mAh cm^{-2}, the functionalized sugarcane chip still has good absorption ability for liquid electrolyte (1 M of $LiPF_6$ in ethylene carbonate/dimethyl carbonate). The liquid electrolyte drops were fully absorbed within 55 s, without liquid leakage.

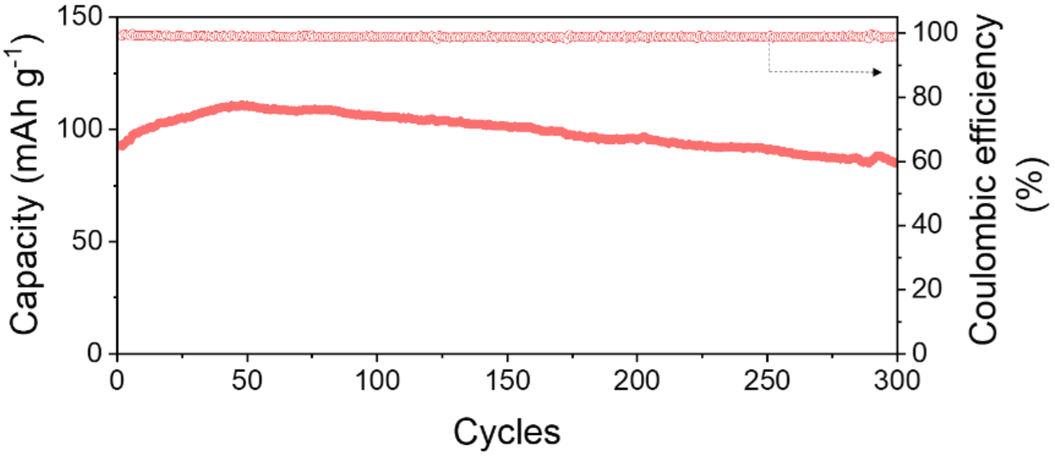

Figure S32. Long-term cycling performance of Sug-Li||Sug-LFP at C-rate of 2C with LFP loading mass of 6 mg cm^{-2}. After 300 cycles, the capacity retains 91 % of its initial value.

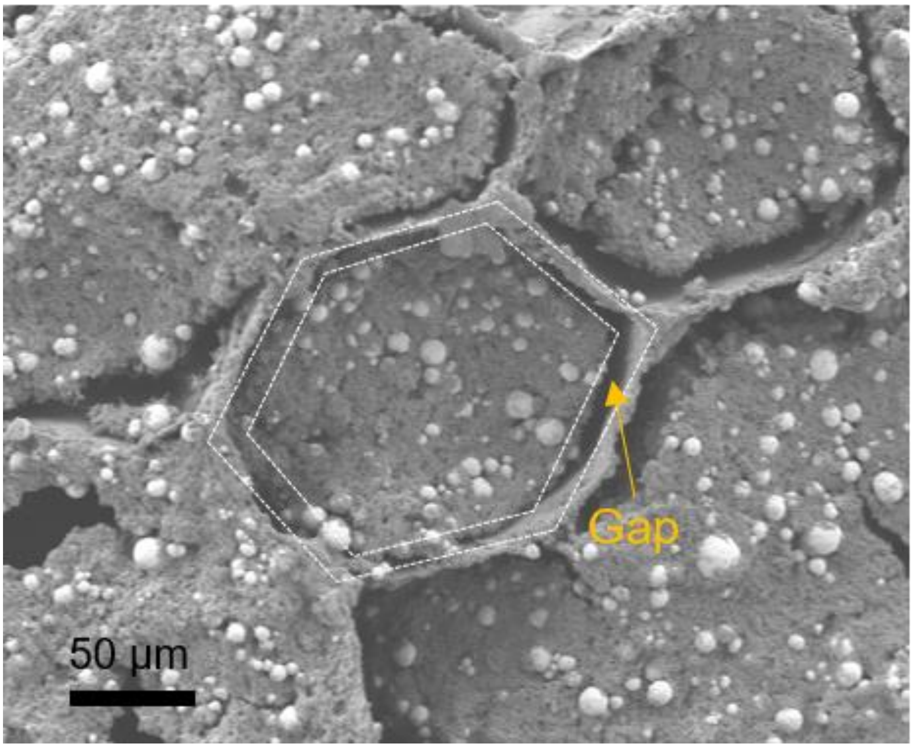

Figure S33. SEM image of Sug-Li||Sug-LFP with loading mass of 43 mg cm^{-2} by self-absorption slurry process. With the evaporation of solvent in slurry, the filled LFP cathode shrank, leaving narrow gap between LFP pillar and honeycomb wall. Note that such gap may facilitate ion transport into the inner space of thick LFP cathode.

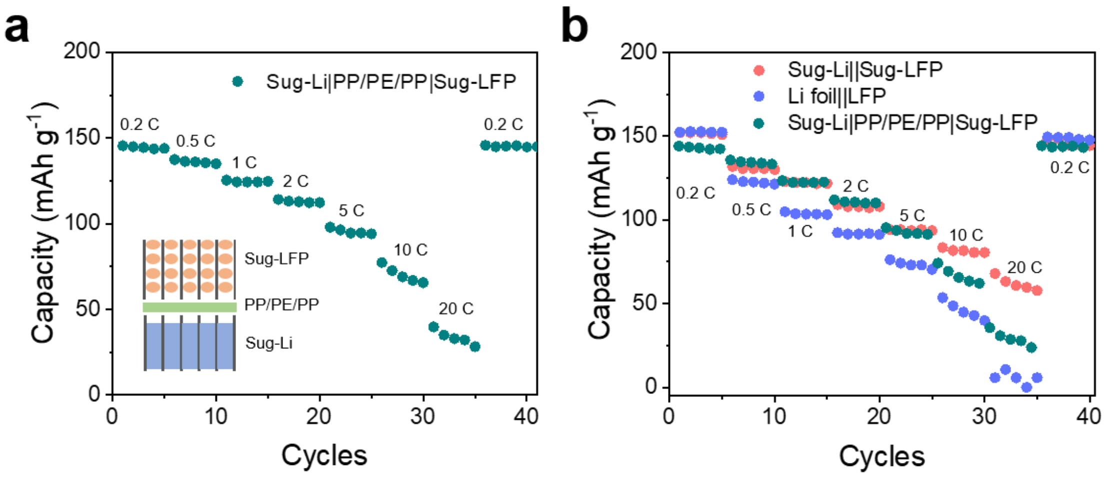

Figure S34. (**a**) Rate performance of assembled cell consisting of cathode Sug-LFP, anode Sug-Li and PP/PE/PP membrane, noted as Sug-Li|PP/PE/PP|Sug-LFP. Inset shows the corresponding structural model. (**b**) Compared with the all-in-one structured Sug-Li||Sug-LFP, Sug-Li|PP/PE/PP|Sug-LFP shows comparable capacity at low C-rate but performs worse at high C-rate.

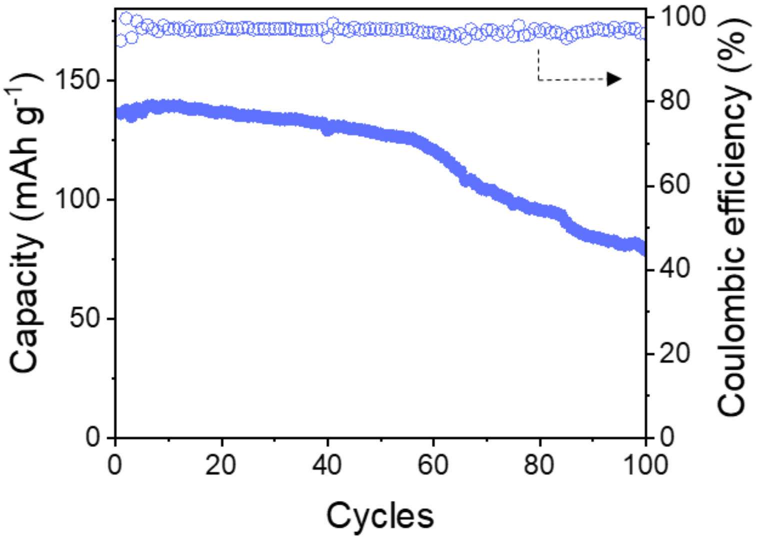

Figure S35. Cycling stability of Li foil||LFP cell counterpart at 0.1 C with LFP loading of 45 mg cm^{-2}.

Table S1. Li-ion diffusion coefficients (D_{Li}^{+}) and Warburg factor (σ_w) of Sug-Li||Sug-LFP with different LFP loading mass.

Cathode (LFP) loading mass (mg cm^{-2})	σ_w	D_{Li+} (cm^2 s^{-1})
6	46	5.3×10^{-14}
9	52.8	4.0×10^{-14}
17	46.5	5.2×10^{-14}
43	64.4	2.7×10^{-14}
76	30.6	1.2×10^{-13}
Li foil||LFP counterpart	210	2.5×10^{-15}

References

[1] B. Wang, Y. Wang, Y. Peng, X. Wang, J. Wang, J. Zhao, *J. Power Source*, **2018**, *390*, 186-196.

[2] A. Mandal, D. Chakrabarty, *Carbohyd. Polym.* **2011**, *86, 1291-1299.*

[3] L. Dong, Z. Chen, S. Lin, K. Wang, C. Ma, H. Lu, *Chem. Mater*. **2017**, *29*, 564-572.

[4] L. Dong, J. Yang, M. Chhowalla, K. P. Loh, *Chem. Soc. Rev*. **2017**, *46*, 7306-7316.
